# Investigating microstructure evolution of lithium metal during plating and stripping via operando X-ray tomographic microscopy

**DOI:** 10.1038/s41467-023-36568-z

**Published:** 2023-02-15

**Authors:** Matthew Sadd, Shizhao Xiong, Jacob R. Bowen, Federica Marone, Aleksandar Matic

**Affiliations:** 1 Department of Physics, Chalmers University of Technology, SE 412 96 Göteborg, Sweden; 2 Xnovo Technology ApS, Galoche Alle 15, 1st Floor, 4600 Køge, Denmark; 3 Paul Scherrer Institute, Swiss Light Source, 5232 Villigen PSI, Switzerland

**Keywords:** Batteries, X-ray diffraction, Imaging studies, Materials for energy and catalysis, Energy storage

## Abstract

Efficient lithium metal stripping and plating operation capable of maintaining electronic and ionic conductivity is crucial to develop safe lithium metal batteries. However, monitoring lithium metal microstructure evolution during cell cycling is challenging. Here, we report the development of an operando synchrotron X-ray tomographic microscopy method capable of probing in real-time the formation, growth, and dissolution of Li microstructures during the cycling of a Li||Cu cell containing a standard non-aqueous liquid electrolyte solution. The analyses of the operando X-ray tomographic microscopy measurements enable tracking the evolution of deposited Li metal as a function of time and applied current density and distinguishing the formation of electrochemically inactive Li from the active bulk of Li microstructures. Furthermore, in-depth analyses of the Li microstructures shed some light on the structural connectivity of deposited Li at different current densities as well as the formation mechanism of fast-growing fractal Li microstructures, which are ultimately responsible for cell failure.

## Abstract (web summary)

Understanding the plating and stripping behaviours of lithium metal is crucial for high-energy battery development. Here, authors track these electrochemical processes in real time by an operando synchrotron X-ray tomographic microscopy, revealing the formation of inactive lithium microstructures.

## Introduction

Li metal is considered as the most important negative electrode active material for Li-based batteries because of its high theoretical specific capacity of 3860 mAh g^−1^, which is an order of magnitude higher than the currently used graphite, and by being the most electropositive metal. When coupled with high-capacity cathodes, either Li insertion materials or conversion chemistries, or applied in a solid-sate configuration, a leap in energy density can be obtained^1–5^. The main challenge in the direct utilization of Li metal as anode is the formation of a mossy and dendritic morphology upon cycling manifested in a low Columbic efficiency and a continuous consumption of the electrolyte through parasitic side reactions, continuous growth of the solid electrolyte interphase (SEI) and irreversible capacity loss on the anode^6–8^. These issues are a result of processes taking place both during charge (Li plating) and discharge (Li stripping), creating an unstable electrode structure.

During cell charge, nonuniform plating leads to the formation of a sparse morphology and the growth of dendritic structures, where rapid growth of certain Li microstructures can cause internal short circuit or even thermal runaway of the cell^9,10^. During discharge, the stripping of Li can result in a collapse of the unstable microstructures produced during plating with the formation of Li domains encapsulated by the electronically insulating SEI and thereby electronically disconnected from the bulk electrode and without electrochemical activity, known as inactive or “dead Li”^11^. Thus, the structural connectivity and architectural stability of the Li metal anode during cycling are critical in order to avoid the formation of inactive Li, dominating the Coulombic efficiency of Li anodes for most electrolytes^11–13^. Therefore, understanding the origin and evolution of Li microstructures during charge and discharge is of great importance to guide the utilization of Li metal anodes in next-generation batteries.

Several strategies have been designed to regulate the microstructure of Li during cycling and to mitigate the formation of inactive Li. These strategies can be sorted into four main categories: (i) electrolyte formulations to obtain a columnar microstructure with low tortuosity^11,14,15^, (ii) three-dimensional host structures to reduce the local current density^16,17^, (iii) artificial SEI to prevent side reactions and to accelerate the interfacial transport of Li ions^18–21^, (iv) tuning operating conditions, such as stack pressure or elevated temperature^22,23^. However, the formation of an ideal microstructure of Li during cycling for a Coulombic efficiency beyond 99.9%, which is a benchmark to support long cycling of practical Li metal batteries, has not been realized yet^24^. Both experimental and computational results show that the final microstructures of plated Li are determined by multiscale processes including reduction of Li-ion, electrocrystallization of Li atoms at the surface, growth of Li nuclei and mass transfer kinetics^25–29^. Thus, tracking these processes at relevant length scales and with a proper temporal resolution, particularly under practical operating conditions, is the key groundwork to understand the formation and evolution of Li microstructures.

A range of tools has been applied to capture the structure of Li metal anodes after cycling, including cryo-electron microscopy^11,30,31^ and magnetic resonance imaging^32^, revealing the nanostructure, including the SEI and chemical changes. With cryogenic focused ion beam–scanning electron microscopy (cryo-FIB–SEM) it has also been possible to in 3D dissect cycled negative electrodes to reveal the inner microstructures after plating^11^.

However, tracking the evolution of the Li microstructures during electrochemical plating and stripping in real time requires an operando experimental setup^33^. 2D optical microscopy has been used to follow Li deposition and dendrite growth^34,35^, but the development of three-dimensional structures and the identification of the process for formation of dead Li, cannot be accessed by this method.

X-ray tomographic microscopy (XTM) is a powerful analytical tool to probe local material morphology as well as composition by nondestructive 3D imaging with a spatial resolution down to sub-micron scale over an extended volume^36^. Because of these advantages, XTM has been extensively used to follow the development of electrode microstructures in a range of battery technologies, including Li-ion, Li-S and Li-air^37–39^. It has also been demonstrated for imaging metallic Li microstructures in cells with liquid as well as solid electrolytes^16,40–44^. However, the contrast for Li metal in attenuation-based tomography is usually insufficient due to the low X-ray attenuation coefficient for Li^45^. This brings challenges to operando experiments when an adequate resolution needs to be balanced with fast acquisition^46,47^. Therefore, previous XTM experiments commonly distinguish Li indirectly by contrasting toward structures with heavier elements, through the formation of voids or cracks, or with respect to a separator defining the interface^48–50^. Under such conditions, details of the Li microstructure are difficult to separate from surrounding components, and thus quantitative analysis is not possible^47^. So far there has been no operando XTM experiment that can directly and continuously track the evolution of Li microstructure in real time during electrochemical plating and stripping.

**Fig. 1:**
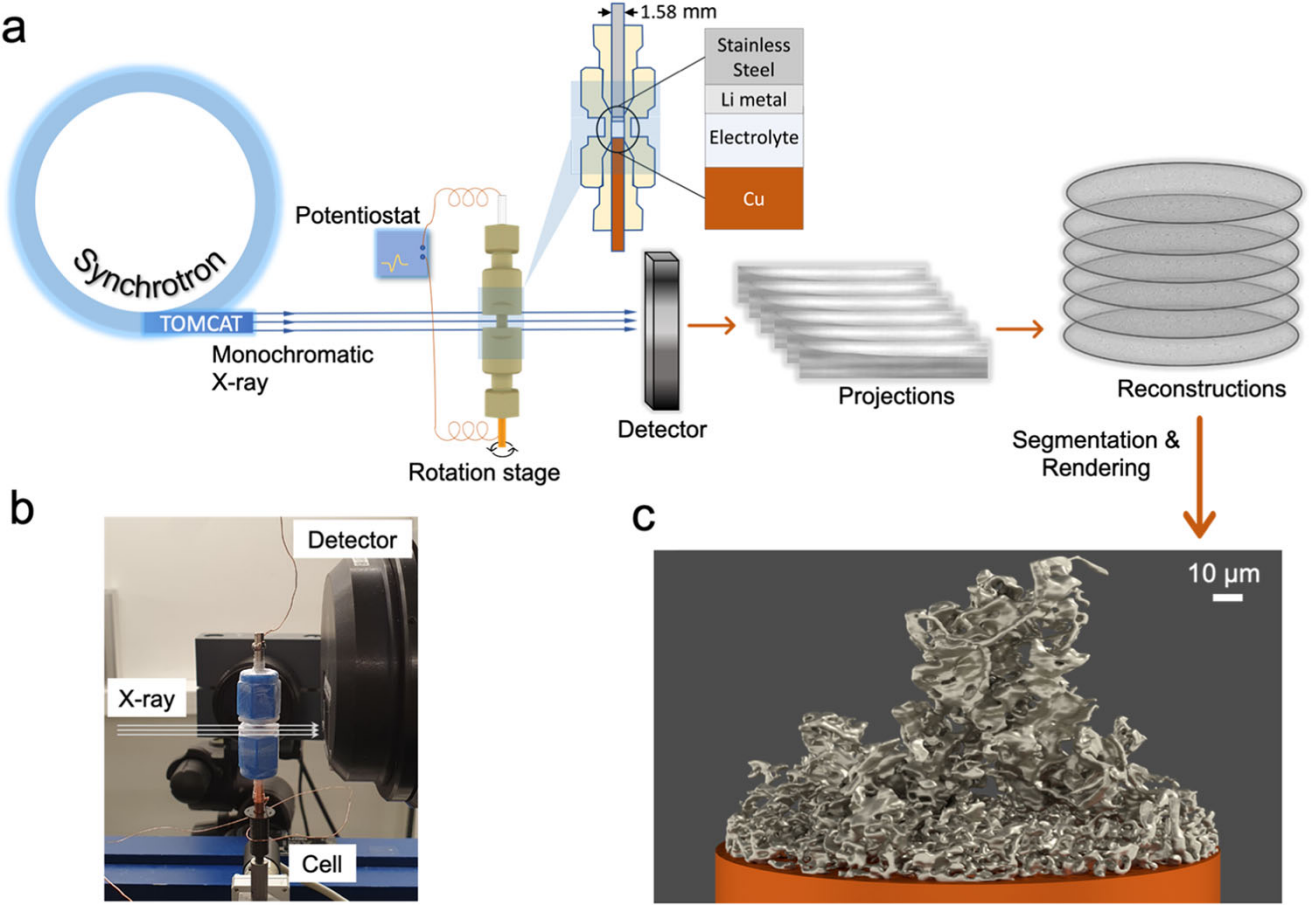
Operando X-ray tomographic microscopy. **a** Illustration of the operando setup, collection of projections and reconstruction. **b** Photographic picture of the experimental setup with the in-house developed operando cell. **c** Example of a 3D visualization of dendritic Li microstructure obtained from X-ray tomographic microscopy measurements.

In this work, we demonstrate the use of operando synchrotron XTM to capture the formation, growth, and dissolution of Li microstructures, as illustrated in Fig. 1a. A dedicated electrochemical cell (Fig. 1b and Supplementary Fig. 1) with a Li|electrolyte|Cu configuration, was designed to carry out operando galvanostatic measurements. The minimized diameter of cell and high flux synchrotron X-ray beam enable a fast acquisition of tomograms, 63 s for full tomogram, with enhanced contrast between Li and surrounding components. All tomograms were collected within a field of view of 0.8 mm and a voxel size of 0.325 μm, resulting in a resolution around 1 μm. The proper segmentation of Li phases of the reconstructed tomograms enabled us to follow the evolution of individual microstructures during plating/stripping of Li. Benefitting from the above advantages we can successfully identify Li, quantitatively track the spatial distribution of deposited Li and its time evolution and capture the formation of dead Li during plating and stripping.

## Results and discussion

### Operando X-ray tomographic microscopy for Li plating and stripping

Operando XTM experiments were performed at the TOMCAT beamline at the Swiss Light Source (SLS). In the experiment, a monochromatic X-ray beam (18 keV) impinges on the operando cell and projections are recorded as the cell is rotated 180° (Fig. 1a, b). 3D images of the microstructures of Li deposited on the Cu working electrode are built up from a set of slices, containing attenuation data for each point in the field of view, reconstructed from recorded projections. The X-ray attenuation is given by Beer–Lambert law^51^:

1 $$I(d)={I}_{0}{e}^{-\mu d}$$

Where *I(d)* and *I*_0_ are the transmitted and incident X-ray intensities, respectively, *μ* is the attenuation coefficient (Supplementary Fig. 1) and *d* is the sample diameter.

The transmission *I/I*_*0*_ should be approximately 13.5% to obtain projections with good attenuation contrast, but this value is usually about 30% or even higher for battery cells^45^. Thus, the sample diameter (*d*) should be minimized to enhance the contrast for the imaging of Li and the diameter of the operando cell was reduced to 1.58 mm and the X-ray energy was set to 18 keV to optimize flux and to enlarge the imaginary part of the index of refraction (*β*). The contrast between Li (low atomic number) and surrounding components (*β*_*sc*_), such as electrolyte as well as side-reaction products forming the SEI (higher atomic number), is determined by

2 $$\triangle \beta={\beta }_{sc}-{\beta }_{Li}$$

As an example of the ability to distinguish Li from other materials in the cell, a projection of cell is shown in Supplementary Fig. 1 and a representative 3D rendering of plated Li on a subsection of the copper substrate is shown in Fig. 1c.

### Evolution of Li microstructures

**Fig. 2:**
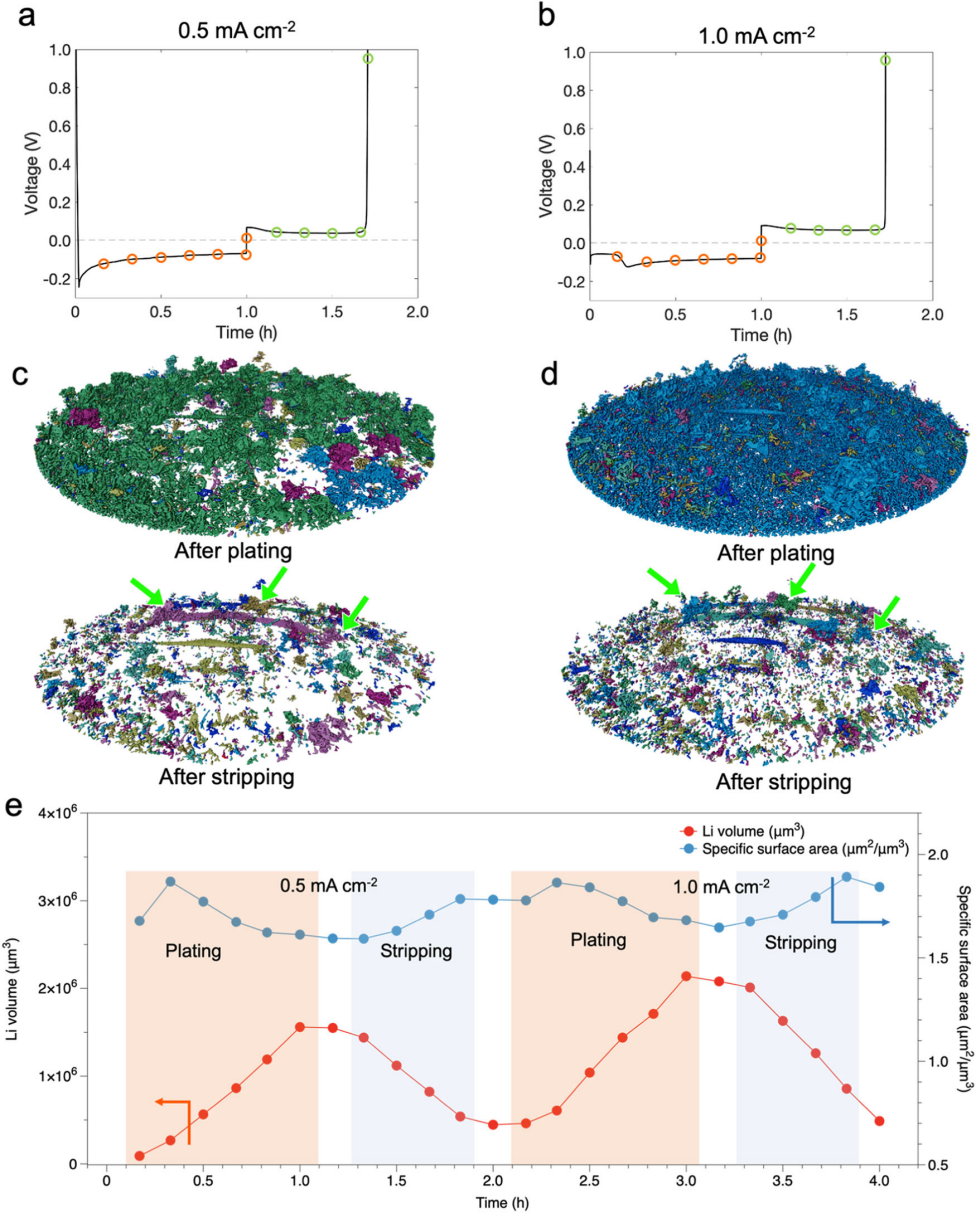
Renderings of the segmented Li from operando X-ray tomograms. **a**, **b** Voltage profiles of galvanostatic plating/stripping during operando XTM at 0.5 mA cm^−2^ for the first cycle (**a**) and at 1.0 mA cm^−2^ in the second cycle (**b**). Circles indicate where X-ray tomograms were taken, and orange color represents plating process while green color represents stripping process. **c**, **d** 3D renderings of segmented Li and identification of isolated regions, rendered as distinct colors. Rendering of tomograms taken after plating and after stripping at 0.5 mA cm^−2^ (**c**) and 1.0 mA cm^−2^ (**d**). Green arrows indicate regions of inactive Li. Various areas of dark blue, light blue, dark green, light green and purple are corresponding to different separated regions of Li. **e** Volume and surface area of deposited Li during plating/stripping cycles. Volume is calculated from all Li voxels in the tomogram and specific surface area exposed to liquid electrolyte. The white areas between colored areas indicate that the tomograms were taken after plating or stripping process.

The results from operando XTM plating/stripping experiments are shown in Fig. 2. The electrolyte used in the experiment was 1.0 M LiTFSI in DOL/DME, a standard non-aqueous electrolyte formulation for Li-based batteries^52–54^ (lithium bis(trifluoromethanesulfonyl)imide (LiTFSI) in 1,2-dimethoxyethane (DME)/1,3-dioxolane (DOL) mixed solvent with a ratio of 1/1 v/v). Galvanostatic voltage profiles during electrochemical plating and stripping at 0.5 mA cm^−2^ (first cycle) and 1.0 mA cm^−2^ (second cycle) are shown in Fig. 2a, b, respectively. The voltage dip at the beginning of Li plating at 0.5 mA cm^−2^ is attributed to the overpotential for initial nucleation of Li on the bare Cu surface^55^. In the second cycle, at higher current density, a smaller overpotential for nucleation is observed, and in addition, a signature of a second nucleation process is present around 15 min into discharge. This behavior cannot be directly related to the higher current density, since in the second cycle there can also be impacts from additional nucleation sites or differences in the SEI compared to uncycled Cu electrode surface in the first cycle. The two nucleation processes are also observed in consecutive cycles of the operando cell at constant current densities (Supplementary Fig. 2) pointing to the difference in voltage profile when depositing on an uncycled surface with respect to the deposition in the second cycle at high current density compared to the behavior at a lower current density where the voltage profiles in the first and second cycle are very similar. X-ray tomograms were taken every 10 min during plating and stripping. Thus, six datasets were collected for plating during 1 h, whereas four datasets were collected during stripping, before the cell polarized and the cut-off voltage of 1.5 V was reached. Tomograms were also acquired after the completion of each plating and stripping.

Reconstruction of the datasets provides 3D renderings of X-ray tomograms and visualization of Li phase is realized by segmentation of Li from surrounding components. All 3D renderings of segmented Li are found in Supplementary Figs. 3 and 4, demonstrating the ability to in real time track the evolution of Li microstructures at the micrometer scale. From 3D renderings of the segmented Li phase, we can obtain direct information on structural connectivity of deposited Li as well as the growth mechanism of Li microstructures. Further segmentation of connected regions from the tomograms after plating and stripping are shown in Fig. 2c, d. The segmentation of Li microstructures formed after plating at low current density in the first cycle (Fig. 2c) shows several separated regions (rendered as green, blue, and purple), while Li deposited at high current density in the second cycle (Fig. 2d) is almost entirely connected over the whole field of view (rendered by blue) apart from a few residual inactive microstructures formed in the previous cycle. This demonstrates the improved connectivity of the Li microstructure formed in the second cycle at a high current density, although the connection with the current collector is not explicitly considered here. The renderings also confirm the presence of inactive Li structures after two full cycles (see green arrows in Fig. 2c, d), where the same isolated regions appear. In addition, the segmented Li volume (Fig. 2e) qualitatively shows the evolution of microstructures following plating and stripping. The measured specific surface area exposed to the liquid electrolyte indicates an increase at the beginning of plating (10–20 min), suggesting a fast growth of fresh surface at this stage. Subsequently, a decreased specific surface area with the deposition of Li can be attributed to the integration of Li microstructures.

### Growth and dissolution of Li dendrites

**Fig. 3:**
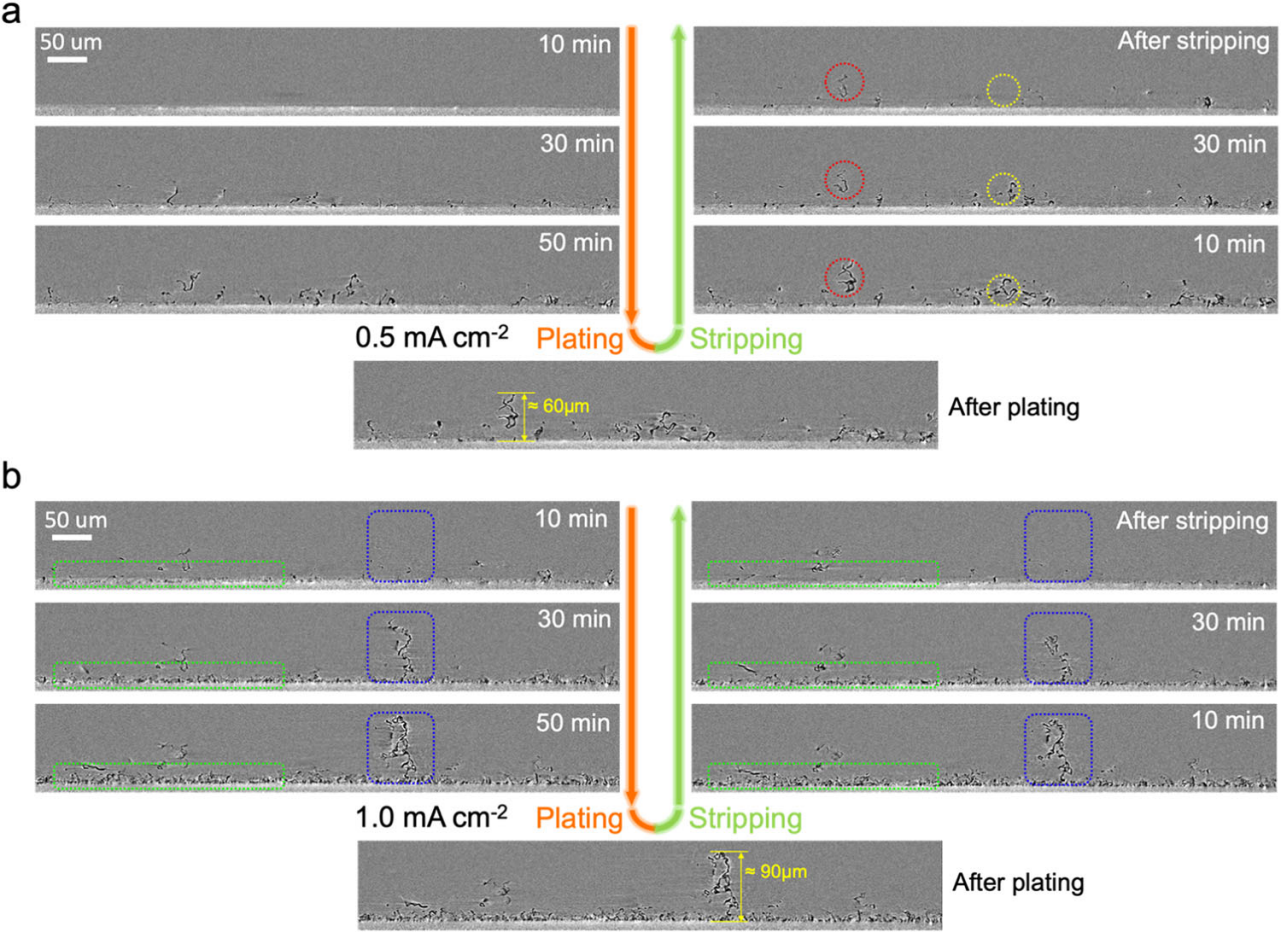
Operando XTM during plating and stripping at different current densities. **a** Reconstructed vertical slices during the first cycle (0.5 mA cm^−2^). Structures with dark color are metallic Li and gray background is the liquid electrolyte (1 M LiTFSI in DOL/DME). **b** Reconstructed vertical slices during the second cycle (1.0 mA cm^−2^).

To directly track the evolution of certain Li microstructures, cross-sectional vertical slices from the same position in the reconstructed 3D tomographic datasets were extracted. They contain part of the Cu substrate, Li deposited on the Cu surface and liquid electrolyte, as shown in Supplementary Fig. 5. All cross-sectional slices for each tomogram are collected in Fig. 3a, b and Supplementary Figs. 6 and 7. Together these slices generate a real-time sequence corresponding to the electrochemical processes of plating and stripping of Li. There is a clear contrast between Li (black/dark regions), the Cu substrate (light gray) and the liquid electrolyte (gray) in the images. In the first cycle of plating at 0.5 mA cm^-2^, we observe a needle-like microstructure as the main form of deposited Li, with a height reaching up to 60 μm after 1-h plating (Fig. 3a). The preferred growth of long Li needles at low current density can be attributed to the preferential deposition of Li at kinks in this needle-like structure^56^. Following the evolution of these structures during stripping, some of them are still electrochemically active (marked by yellow dashed circles in Fig. 3a) and almost totally disappear as stripping proceeds. In contrast, the Li microstructure marked by red dashed circle is only partly dissolved during stripping and nearly half of it remains on the Cu substrate at the end of stripping. Thus, we here directly observe the generation of inactive Li and previous literature suggest that disconnection from the electronically conductive Li network is the main reason for the formation of inactive Li during stripping^11,22,24^.

When increasing the current density to 1.0 mA cm^–2^ in the second cycle, rapid growth of a mossy-like microstructure (marked by green dashed rectangles in Fig. 3b) is observed, between 10 and 30 min into plating, which is consistent with the second nucleation signature in the voltage profile in Fig. 2b. The layer of mossy-like Li is approximately 10-μm thick and it is highly reversible upon stripping, see the slice after full stripping (Fig. 3b). Another significant feature observed during plating at high current density is the growth of a large Li microstructure (marked by blue dashed rectangles in Fig. 3b), combining both mossy and needle-like morphologies, that reach a 90 μm height after 1 h of plating. This type of Li microstructure is considered as the most dangerous morphology since the fast vertical propagation and small branches can easily penetrate a porous separator in a cell, leading to an internal short circuit^8,34^.

**Fig. 4:**
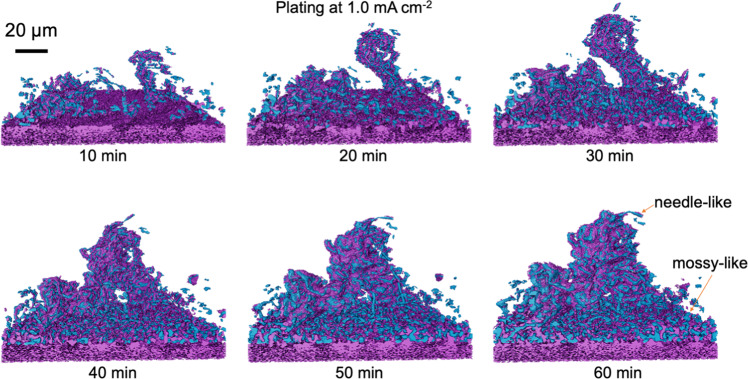
Evolution of Li microstructure formed by the integration of a Li dendrite and mossy structures. Renderings are extracted from tomograms taken during plating at 1.0 mA cm^–2^ for 10, 20, 30, 40, 50, and 60 min. Li is shown in blue, purple covering Li is SEI or high atomic number components, and purple in the bottom is Cu substrate.

To track the formation process of this crucial Li microstructure, the focused region is extracted from the reconstructed 3D tomograms to follow the integration of mossy and needle-like Li microstructures. As shown in Fig. 4, the metallic Li is segmented and rendered in blue and the components rendered in purple are Cu substate (purple part at bottom) or SEI components (purple part covering the Li) with higher atomic number^41^. During plating at 1.0 mA cm^–2^, an isolated Li cluster (to the right in the rendering) rapidly grows, with an increased risk of collapse as its length increases. Simultaneously, the surrounding mossy-like Li extends to cover the substrate surface and grows in thickness. After 40 min of deposition, the isolated Li cluster makes contact with the mossy base, forming an integrated architecture with enhanced mechanical integrity. Therefore, this integrated Li microstructure that combines needle-like Li and mossy-like Li is particularly hazardous with respect to the penetration of a separator and the creation of a short circuit during the charging process of the battery.

### Spatial distribution of deposited Li

**Fig. 5:**
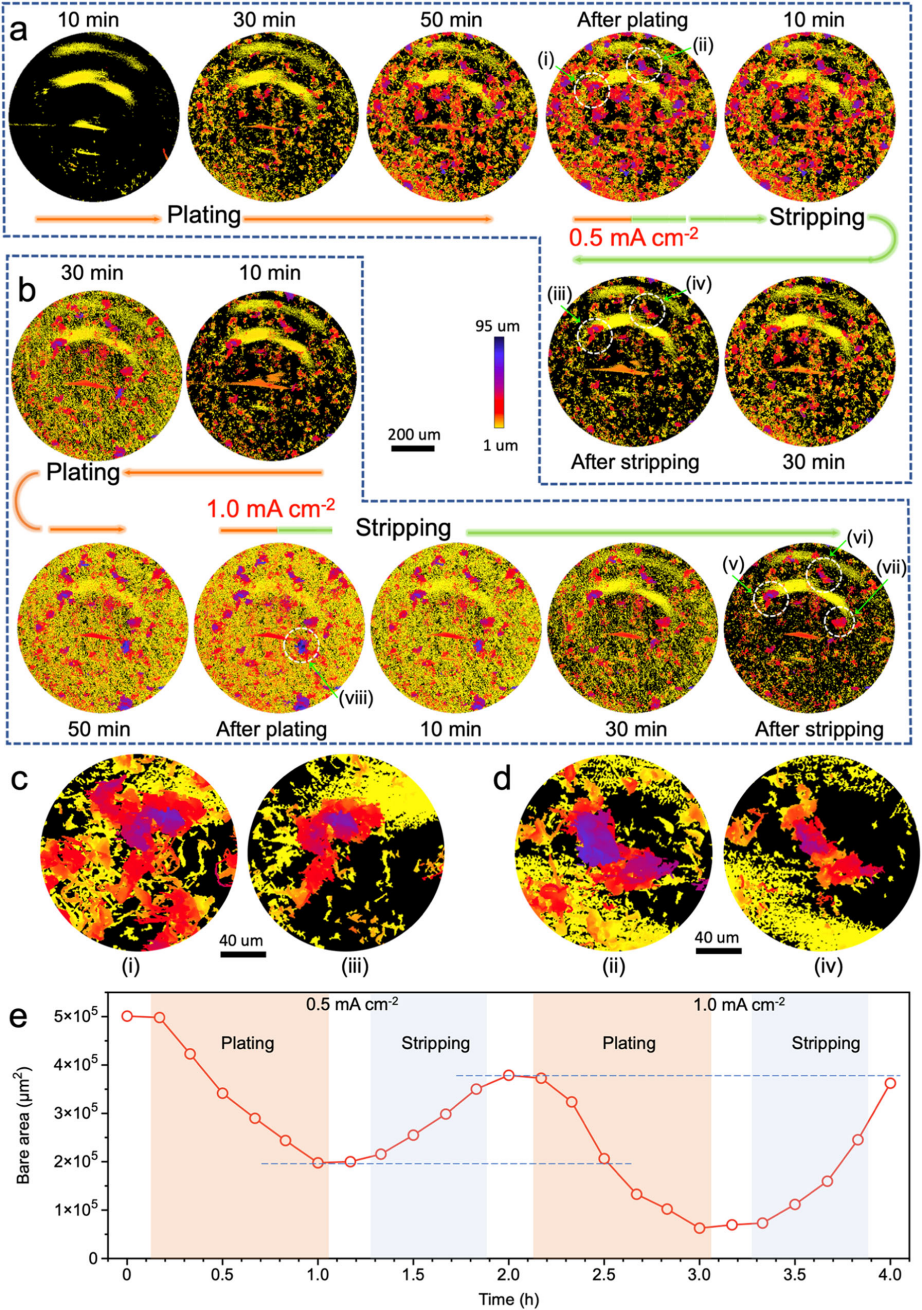
Height maps of deposited Li at different stages of plating and stripping. **a**, **b** Height maps of Li deposited at 0.5 mA cm^–2^ in the first cycle (**a**) and at 1.0 mA cm^−2^ in the second cycle (**b**), respectively. The height of Li projected on one pixel (0.325 μm × 0.325 μm) on the Cu surface is visualized by the color scale bar. The black pixels are bare Cu substrate. Typical Li microstructures identified as electrochemically inactive are marked by white dashed circles and labeled from (i) to (viii). **c**, **d** Close up of inactive Li microstructures marked in maps after plating and after stripping at 0.5 mA cm^–2^ in **a**. **e** Evolution of bare area on the Cu substrate during plating/stripping cycles at 0.5 and 1.0 mA cm^–2^. The white areas between colored areas are corresponding to the tomograms taken after plating or stripping process.

The cross-sectional images or focused region only sample one area of the full X-ray tomogram. In Fig. 5, a height map of all Li microstructures within the full field of view (0.8 mm × 0.7 mm) shows the overall microstructure evolution during plating and stripping. The height of Li microstructures is visualized by showing the orthographic representation of the segmented Li, the height in each pixel on the Cu substrate represented with a color scale bar (Fig. 5a, b). Li microstructures appear as a distribution of islands after plating at 0.5 mA cm^–2^ for 30 min and after 1 h of plating the height is over 50 μm in many locations (purple areas in Fig. 5a and Supplementary Fig. 8). This confirms that needle-like Li is formed over the entire substrate and the structures observed in Fig. 3 are not just formed in a local hotspot.

As expected, the height of Li microstructures decreases during stripping at 0.5 mA cm^−2^, but it is evident that a certain amount of Li remains on the substrate after full electrochemical stripping, potentially being locations for the formation of inactive Li. To track the formation of inactive Li, two of these regions marked by a white dashed circle in Fig. 5a, corresponding to complete plating and complete stripping. Part of the Li microstructure in the local area marked by circles (i) and (iii) of Fig. 5a, enlarged in Fig. 5c, already exists at 50 min of plating, and barely shows any change after that during plating, neither during stripping. This suggests that the disconnection of deposited Li from the electronically conductive network occurs at the very early stages of stripping or even already during plating. The Li microstructure in the circle (ii) and (iv) of Fig. 5a, enlarged in Fig. 5d, shows a decreased height and a decrease in the area covered by Li after stripping. However, a large cluster of inactive Li is left after stripping, suggesting its disconnection from the electronically conductive network during the dissolution of Li^6,11^.

The presence of mossy-like Li observed in Fig. 3b is further demonstrated in the height maps by the high density of low-height microstructures (Fig. 5b and Supplementary Fig. 9), showing them to be the dominant morphology of Li deposited at the higher current density in the second cycle. The local current density on the area of bare Cu (black area) is increased as a result of the fact that part of Cu surface is covered by inactive Li created in the first cycle. It has been previously reported that higher local current density drives the formation of a higher number of nuclei with smaller radii, promoting the growth of mossy-like microstructures^8,35,57^. The high reversibility of the mossy-like microstructure is also proven here as most of it disappears after stripping. In the second plating and stripping cycle at high current density (1.0 mA cm^–2^), one large residual cluster of inactive Li microstructure from the first cycle, region (v) and (vi) in Fig. 5b, and one freshly formed inactive Li microstructure, region (vii) in Fig. 5b, are observed after full stripping. In addition, the large fractal Li microstructure found in Fig. 3b, and Fig. 4 (after plating) is also marked as region (viii) in Fig. 5b (in height map after plating). The dark blue area reveals that its height is close to 100 μm. Surprisingly, most of the highest Li microstructures still seem to maintain some connectivity to the electronically conductive network, as they show partial dissolution during stripping.

The bare area on the Cu substrate is an indicator for the spatial distribution of Li at various stages of plating and stripping. As shown in Fig. 5e, the bare area decreases as plating proceeds and 60% of the Cu surface is covered by Li deposits after plating for 1 h at 0.5 mA cm^–2^. The bare area continuously increases during stripping but only 76% of the initial bare Cu surface is recovered after full stripping, the rest is occupied by inactive Li. A similar trend is observed during plating/stripping at 1.0 mA cm^–2^ in the second cycle. However, the bare area at the start of the second cycle is almost fully recovered at end of stripping (dashed line in Fig. 5e), showing a higher reversibility in the second cycle at high current density. It suggests that the inactive Li generated in the second cycle integrates with the disconnected Li structures generated in the first cycle, partially reactivating some regions on the surface of the substrate.

**Fig. 6:**
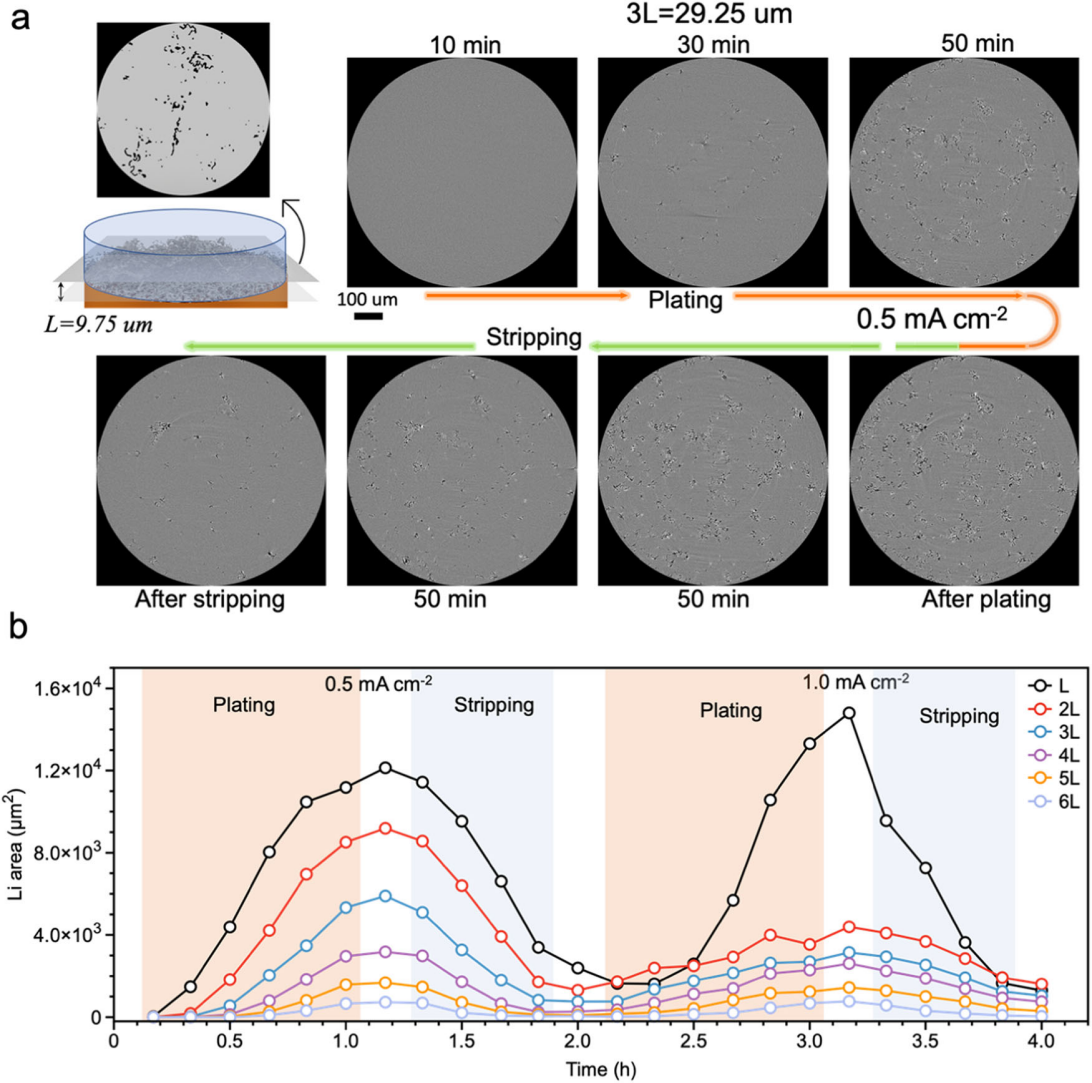
Spatial evolution of Li microstructures during plating and stripping cycles. Horizontal slices at fixed distances, n·L = n·9.75 μm (L represents the total length of 30 voxels, 9.75 μm) from the Cu substrate are taken from each 3D tomogram (1–10 L), same tomogram as those in Supplementary Figs. 3 and 4. **a** Slices taken at 3 L (29.25 μm from the Cu substrate) are shown as an example. Dark features are deposited Li, and gray region is liquid electrolyte. **b** Evolution of area of segmented Li at different distances from the Cu surface (i.e., in each slice) during plating/stripping at 0.5 and 1.0 mA cm^–2^. The white areas between colored areas indicate the tomograms which are taken after plating or stripping process.

To reveal the vertical distribution of Li more precisely, we extracted horizontal 2D slices at fixed distances (n·L = n·9.75 μm, *n* = 2–10) from the Cu substrate and determined the area of Li in each slice from the segmentation of Li pixels, see schematic in Fig. 6a. Here L represents a certain length of 9.75 μm, which is corresponding to the total length of 30 voxels. The segmentation of Li phase is shown in Supplementary Fig. 10, see more details in the methods section (Analysis of reconstructed tomograms) and Supplementary Information (Supplementary Note 1: Method for Segmentation of Li phase). Figure 6a and Supplementary Fig. 11 show slices at a distance of 3 L from the Cu surface during plating/stripping at low current density in the first cycle. These images reveal that the needle-like Li microstructure appears early and continues to grow during plating. The structures are partly reversible during stripping, but residual Li is still observed in the last slice. The evolution of the area covered by Li at different distances from the substrate over the two plating and stripping cycles, with low and high current densities, respectively, is shown in Fig. 6b and Supplementary Fig. 12. Initially the growth is slow, and a negligible amount of Li is found close to the substrate (L) after plating at 0.5 mA cm^−2^ for 10 min and subsequently the amount of Li  gradually increases in all slices during plating. In contrast, in the second cycle at high current density there is mainly an increase of the covered area in the slice closest to the substrate (L), whereas the increase at 2L–4L is smaller than in the first cycle. At slightly larger distances (5 and 6 L) the covered area is similar to that in the first cycle. However, one can also observe that the amount of extended structures is not close to the substrate, at distances ≥8 L (Supplementary Fig. 12). This quantitative analysis demonstrates the low height of mossy-like Li microstructures in contrast to the extended needle-like Li morphology in the first cycle, and the coexistence of mossy and needle-like microstructures in the second cycle.

In summary, 3D visualization of the microstructure evolution of Li during electrochemical plating and stripping in a non-aqueous liquid electrolyte solution has here been demonstrated by operando XTM. A spatial resolution of ~1 µm allows us to distinguish three typical Li microstructures, needle-like, mossy-like, and integrated structures, whose presence is strongly related to the applied current density. Analysis of height distribution of Li demonstrates that tall needle-like structures are formed at low current density, whereas shorter and denser mossy-like Li is formed at high current density coexisting with tall needle-like Li. We identify the integration of these structures as the mechanism of mechanically robust and fast-growing Li dendrites. The formation of inactive Li, which is one of the main reasons for low Coulombic efficiency of Li anodes, is also directly captured by tracking the evolution of Li structures during consecutive plating and stripping cycles. We show that the generation of inactive Li occurs not only during stripping due to structural collapse and encapsulation by SEI, but also during plating as a result of the fast formation of SEI, cutting the connection to the electronic conductive network. The structural connection of deposited Li is further revealed by the segmentation of isolated regions.

We are able to determine geometry, connectivity and electrochemical activity of Li structures and their dependence on operating conditions, which are all key factors linked to Coulombic efficiency and safety in practical Li metal batteries. The typical Li microstructures captured by the operando XTM here provide an understanding of the design of ideal structures of deposited Li. The needle-like microstructure with low reversibility during stripping can be suppressed to reduce the formation of inactive Li. In contrast, the mossy-like Li microstructures are highly reversible upon stripping. It is worth emphasizing that the large Li microstructure formed by the integration of mossy-like Li and needle-like Li should be avoided since its presence is a great safety hazard for the operation of a battery. Our work demonstrates operando X-ray tomography as a nondestructive technique to quantitatively track the evolution of internal microstructures of Li in real time. In future work, it would be of interest to image Li microstructures operando at higher current densities mimicking more realistic operating conditions. This would require even shorter acquisition time to avoid blurring of the images in operando experiments and can be achieved by optimizing the experimental conditions with decreased integration time per projection, reducing the number of projections and through cell design. The technique could also be applied to investigate the evolution of microstructures in general for metal anodes, such as in sodium (Na), zinc (Zn), or magnesium (Mg) batteries.

## Methods

### Electrolyte preparation

LiTFSI (99.9%, H_2_O < 20 ppm, SOLVIONIC) was dried at 80 °C under vacuum for 24 h in a Büchi oven inside an argon-filled glovebox (H_2_O content <0.1 ppm, O_2_ content <0.1 ppm) before use. DME (purity ≥99.5%, Sigma Aldrich) and DOL (purity ≥99.8%, Sigma Aldrich) were dried over molecular sieves (3 Å) for 24 h before use. The liquid electrolyte was prepared by dissolving 1.0 M LiTFSI in a mixture of DOL and DME with a 1:1 volume ratio. All materials were stored and handled inside an argon atmosphere glovebox (H_2_O content <0.1 ppm, O_2_ content <0.1 ppm).

### Cell fabrication

A dedicated electrochemical cell for operando XTM was built on a polymeric tube fitting with a 1.58 mm internal diameter. The cell material is polyether ether ketone which minimizes the X-ray attenuation while providing good sealing for the electrodes and the electrolyte. The middle section of the tube fitting was cut to reduce the outer diameter of the cell and the electrodes were mounted so that the electrode surfaces were within this area of the cell, as shown in Fig. 1 and Supplementary Fig. 1. A stainless-steel pin (1.58 mm in diameter) with a Li foil (200 μm in thick, 3.5 cm in wide, purity >99.8%, Honjo Metals) attached to the surface was used as the Li counter electrode and a Cu pin (K&S metals, 99.9% purity, 1.58 mm in diameter, 4 cm in length) with same diameter was employed as working electrode for the electrochemical plating/stripping of Li. The Cu pin was polished to obtain a flat substrate, washed by acetone (VWR Chemicals) and isopropanol (VWR Chemicals) in an ultrasonic bath for 30 min and dried at 80 °C for 24 h under a vacuum to avoid oxidation. Cell assembly was performed in the argon-filled glovebox (H_2_O content <0.1 ppm, O_2_ content <0.1 ppm). A stainless-steel pin with Li was first pushed into the cell and held in place by the casing’s compression fitting assembly. 10 μL electrolyte was added into the cell and the Cu pin was pushed in without a separator and sealed in place.

### Operando synchrotron X-ray tomographic microscopy

Operando XTM experiments were performed at the TOMCAT Beamline (X02DA) at the SLS, Paul Scherrer Institute, Switzerland. The X-ray energy was 18 keV, a compromise between enhanced attenuation contrast of Li, limited radiation damage and high flux. A 20 µm thick LuAg:Ce scintillator was used to convert the X-ray beam to visible light which was subsequently magnified by a ULAPO20× objective. The images were acquired by a PCO.Edge camera (2560 × 2160 pixels) which gives a 0.325 µm pixel size and a total field-of-view of 0.8 × 0.7 mm^2^. A total of 1000 projections were taken for the acquisition of each tomogram, equally distributed over 180 degrees, with an exposure time of 50 ms and an overhead time of 13 ms. Thus, the total time for recording each tomogram was 63 s. A 100 µm Al filter and a 50 µm Al filter were introduced to reduce the X-ray flux to reduce radiation damage of the electrolyte. Tomographic reconstruction of the projections was performed using the Gridrec reconstruction routine^58^.

The galvanostatic cycling under different current densities was conducted by using a Gamry Reference 600 potentiostat at a room temperature of 20 °C. The cut-off conditions for electrochemical plating and stripping are 1 h and 1.5 V, respectively. X-ray tomograms were taken before galvanostatic operation, every 10 min during the electrochemical operation, and after each plating/stripping step.

### Analysis of reconstructed tomograms

All reconstructed images had a median filter applied to decrease noise. Segmentation of the Li phase was subsequently performed using thresholding, where the threshold value was chosen via visual inspection. Details for the segmentation of Li phases are found in Supplementary Information (Supplementary Note 1 and Supplementary Figs. 13−21). The isolated regions of Li in Fig. 2 were determined by using the Label Analysis module of the AVIZO software package on the segmented Li volumes. Isolated regions are individual segmented Li structures that are not connected to an adjacent region via a segmented voxel.

The purple region seen in Fig. 4 is a further segmentation of the reconstructed volume. The same process is followed as for the segmentation of the Li regions, but with a different range of grayscale compared to the Li phase.

The area of Li in Fig. 6 is determined through a segmentation procedure of the black voxels in an individual slice. The number of voxels that are assigned to Li are then multiplied by the surface area of a voxel (0.325 µm × 0.325 µm) to give the area of Li in a specific slice.

The calculation of the object-specific surface of the Li microstructures in Fig. 2e is carried out using the Object Specific Surface module of AVIZO software in which the object-specific surface (µm^2^/µm^3^) of the 3D case is calculated using the following equation:

3 $$Object\,Specific\,Surface=\frac{\left({area}3d\right)}{\left({volume}3d\right)}$$

${area}3d$ is a module in AVIZO that calculates ‘the area of the object boundary’ which takes into account ‘the exposed surfaces of outer voxels’. Thus, ${area}3d$ provides a value in µm^2^ of the exposed Li surface area of binarized Li voxels. ${volume}3d$ is calculated as the volume of all binarized voxels of Li.

The height maps were created from orthographic projections, with the color bar representing the height of the segmented Li phase.

3D rendering of the segmented Li phase was performed using AVIZO, along with the volume analysis which calculated the volume by multiplying the number of segmented Li voxels, by the voxel volume (0.325 µm × 0.325 µm × 0.325 µm). The Li phase-specific surface area was then calculated by dividing the surface area of the Li phase by the Li volume.

### Reporting summary

Further information on research design is available in the Nature Portfolio Reporting Summary linked to this article.

## Supplementary information

Supplementary Information

Peer Review File

Reporting Summary

## Data Availability

Raw reconstructed images for all tomograms, including orthographic projections, horizontal images, vertical slices and volume rendering in this study, have been deposited in the Zenodo repository, 10.5281/zenodo.6364877. Source Data are provided with this paper.

## Source data

Source data

## References

[1] Tarascon JM, Armand M (2001). Issues and challenges facing rechargeable lithium batteries. Nature.

[2] Xue W (2021). Ultra-high-voltage Ni-rich layered cathodes in practical Li metal batteries enabled by a sulfonamide-based electrolyte. Nat. Energy.

[3] Lee Y-G (2020). High-energy long-cycling all-solid-state lithium metal batteries enabled by silver–carbon composite anodes. Nat. Energy.

[4] Varzi A, Raccichini R, Passerini S, Scrosati B (2016). Challenges and prospects of the role of solid electrolytes in the revitalization of lithium metal batteries. J. Mater. Chem. A.

[5] Xu X (2022). Electro–chemo–mechanical failure of solid electrolytes induced by growth of internal lithium filaments. Adv. Mater..

[6] Yoshimatsu I, Hirai T, Yamaki JI (1988). Lithium electrode morphology during cycling in lithium cells. J. Electrochem. Soc..

[7] Liu J (2019). Pathways for practical high-energy long-cycling lithium metal batteries. Nat. Energy.

[8] Wood KN, Noked M, Dasgupta NP (2017). Lithium metal anodes: toward an improved understanding of coupled morphological, electrochemical, and mechanical behavior. ACS Energy Lett..

[9] Cheng XB, Zhang R, Zhao CZ, Zhang Q (2017). Toward safe lithium metal anode in rechargeable batteries: a review. Chem. Rev..

[10] Xu W (2014). Lithium metal anodes for rechargeable batteries. Energy Environ. Sci..

[11] Fang CC (2019). Quantifying inactive lithium in lithium metal batteries. Nature.

[12] Xiao J (2020). Understanding and applying coulombic efficiency in lithium metal batteries. Nat. Energy.

[13] Louli AJ (2020). Diagnosing and correcting anode-free cell failure via electrolyte and morphological analysis. Nat. Energy.

[14] Yu H (2017). Dendrite-free lithium deposition with self-aligned columnar structure in a carbonate–ether mixed electrolyte. ACS Energy Lett..

[15] Zhang XQ (2017). Columnar lithium metal anodes. Angew. Chem. Int. Ed. Engl..

[16] Harry KJ, Hallinan DT, Parkinson DY, MacDowell AA, Balsara NP (2014). Detection of subsurface structures underneath dendrites formed on cycled lithium metal electrodes. Nat. Mater..

[17] Lin D (2016). Layered reduced graphene oxide with nanoscale interlayer gaps as a stable host for lithium metal anodes. Nat. Nanotechnol..

[18] Cao X (2019). Monolithic solid–electrolyte interphases formed in fluorinated orthoformate-based electrolytes minimize Li depletion and pulverization. Nat. Energy.

[19] Kim MS (2018). Langmuir–Blodgett artificial solid-electrolyte interphases for practical lithium metal batteries. Nat. Energy.

[20] Liu, Y. et al. An artificial solid electrolyte interphase with high Li-Ion conductivity, mechanical strength, and flexibility for stable lithium metal anodes. *Adv. Mater*. **29**,1605531 (2017). 10.1002/adma.201605531 28032934

[21] Liu Y (2022). Electro-chemo-mechanical modeling of artificial solid electrolyte interphase to enable uniform electrodeposition of lithium metal anodes. Adv. Energy Mater..

[22] Fang C (2021). Pressure-tailored lithium deposition and dissolution in lithium metal batteries. Nat. Energy.

[23] Wang J (2019). Improving cyclability of Li metal batteries at elevated temperatures and its origin revealed by cryo-electron microscopy. Nat. Energy.

[24] Hobold GM (2021). Moving beyond 99.9% Coulombic efficiency for lithium anodes in liquid electrolytes. Nat. Energy.

[25] Wang X (2020). Glassy Li metal anode for high-performance rechargeable Li batteries. Nat. Mater..

[26] Liu Y (2021). Insight into the critical role of exchange current density on electrodeposition behavior of lithium metal. Adv. Sci..

[27] Xu X (2020). Role of Li‐Ion depletion on electrode surface: underlying mechanism for electrodeposition behavior of lithium metal anode. Adv. Energy Mater..

[28] Zhang R, Shen X, Cheng X-B, Zhang Q (2019). The dendrite growth in 3D structured lithium metal anodes: Electron or ion transfer limitation?. Energy Storage Mater..

[29] Liu Z (2021). Dendrite-free lithium based on lessons learned from lithium and magnesium electrodeposition morphology simulations. Cell Rep. Phys. Sci..

[30] Zachman MJ, Tu Z, Choudhury S, Archer LA, Kourkoutis LF (2018). Cryo-STEM mapping of solid-liquid interfaces and dendrites in lithium-metal batteries. Nature.

[31] Li Y (2017). Atomic structure of sensitive battery materials and interfaces revealed by cryo-electron microscopy. Science.

[32] Chandrashekar S (2012). 7Li MRI of Li batteries reveals location of microstructural lithium. Nat. Mater..

[33] Improving the fundamental understanding of batteries via operando measurements. *Nat. Commun*. **13**, 4723 (2022). 10.1038/s41467-022-32245-9 PMC9378737 35970841

[34] Bai P, Li J, Brushett FR, Bazant MZ (2016). Transition of lithium growth mechanisms in liquid electrolytes. Energy Environ. Sci..

[35] Wood KN (2016). Dendrites and pits: untangling the complex behavior of lithium metal anodes through operando video microscopy. ACS Cent. Sci..

[36] Withers PJ (2021). X-ray computed tomography. Nat. Rev. Methods Prim..

[37] Tan C (2018). Four-dimensional studies of morphology evolution in lithium–sulfur batteries. ACS Appl. Energy Mater..

[38] Sun F (2018). Revealing hidden facts of Li anode in cycled lithium–oxygen batteries through X-ray and neutron tomography. ACS Energy Lett..

[39] Sadd M (2022). Visualization of dissolution-precipitation processes in lithium–sulfur batteries. Adv. Energy Mater..

[40] Sun F (2016). Study of the mechanisms of internal short circuit in a Li/Li cell by synchrotron X-ray phase contrast tomography. ACS Energy Lett..

[41] Eastwood DS (2015). Three-dimensional characterization of electrodeposited lithium microstructures using synchrotron X-ray phase contrast imaging. Chem. Commun..

[42] Sun F (2016). Morphological evolution of electrochemically plated/stripped lithium microstructures investigated by synchrotron X-ray phase contrast tomography. ACS Nano.

[43] Cheng J-H (2017). Visualization of lithium plating and stripping via in operando transmission X-ray microscopy. J. Phys. Chem. C..

[44] Seitzman N (2021). Operando X-ray tomography imaging of solid-state electrolyte response to Li evolution under realistic operating conditions. ACS Appl. Energy Mater..

[45] Pietsch P, Wood V (2017). X-ray tomography for lithium ion battery research: a practical guide. Annu. Rev. Mater. Res..

[46] Adair KR (2020). Temperature-dependent chemical and physical microstructure of Li metal anodes revealed through synchrotron-based imaging techniques. Adv. Mater..

[47] Yu S-H, Huang X, Brock JD, Abruña HD (2019). Regulating key variables and visualizing lithium dendrite growth: an operando X-ray study. J. Am. Chem. Soc..

[48] Lewis JA (2021). Linking void and interphase evolution to electrochemistry in solid-state batteries using operando X-ray tomography. Nat. Mater..

[49] Sun F (2020). Morphological reversibility of modified Li-based anodes for next-generation batteries. ACS Energy Lett..

[50] Ning Z (2021). Visualizing plating-induced cracking in lithium-anode solid-electrolyte cells. Nat. Mater..

[51] Grodzins L (1983). Optimum energies for x-ray transmission tomography of small samples: applications of synchrotron radiation to computerized tomography I. Nucl. Instrum. Methods Phys. Res..

[52] He X (2021). The passivity of lithium electrodes in liquid electrolytes for secondary batteries. Nat. Rev. Mater..

[53] Aurbach D (2009). On the surface chemical aspects of very high energy density, rechargeable Li–sulfur batteries. J. Electrochem. Soc..

[54] Steiger J, Kramer D, Mönig R (2014). Microscopic observations of the formation, growth and shrinkage of lithium moss during electrodeposition and dissolution. Electrochim. Acta.

[55] Yan K (2016). Selective deposition and stable encapsulation of lithium through heterogeneous seeded growth. Nat. Energy.

[56] Steiger J, Kramer D, Mönig R (2014). Mechanisms of dendritic growth investigated by in situ light microscopy during electrodeposition and dissolution of lithium. J. Power Sources.

[57] Biswal P (2021). The early-stage growth and reversibility of Li electrodeposition in Br-rich electrolytes. Proc. Natl Acad. Sci. USA.

[58] Marone F, Stampanoni M (2012). Regridding reconstruction algorithm for real-time tomographic imaging. J. Synchrotron Radiat..

