# Supplementary material for: Investigating microstructure evolution of lithium metal during plating and stripping via operando X-ray tomographic microscopy
Source: Nat Commun. 2023 Feb 15;14:854. doi: 10.1038/s41467-023-36568-z (PMC9931753; doi:10.1038/s41467-023-36568-z)
Supplement: Supplementary file 1 — Supplementary Information [file 41467_2023_36568_MOESM1_ESM.pdf]

Supplementary Information

Investigating microstructure evolution of lithium metal during plating and stripping via operando X-ray tomographic microscopy

Matthew Sadd[1,4], Shizhao Xiong[1,4*], Jacob R. Bowen[2], Federica Marone[3], Aleksandar Matic[1,*]

[1] Department of Physics, Chalmers University of Technology, SE 412 96, Göteborg, Sweden.

[2] Xnovo Technology ApS, Galoche Alle 15,1st Floor, 4600 Køge, Denmark

[3] Paul Scherrer Institute, Swiss Light Source, 5232 Villigen PSI, Switzerland.

[4] These authors contributed equally: Matthew Sadd, Shizhao Xiong.

*E-mail: shizhao.xiong@chalmers.se, matic@chalmers.se

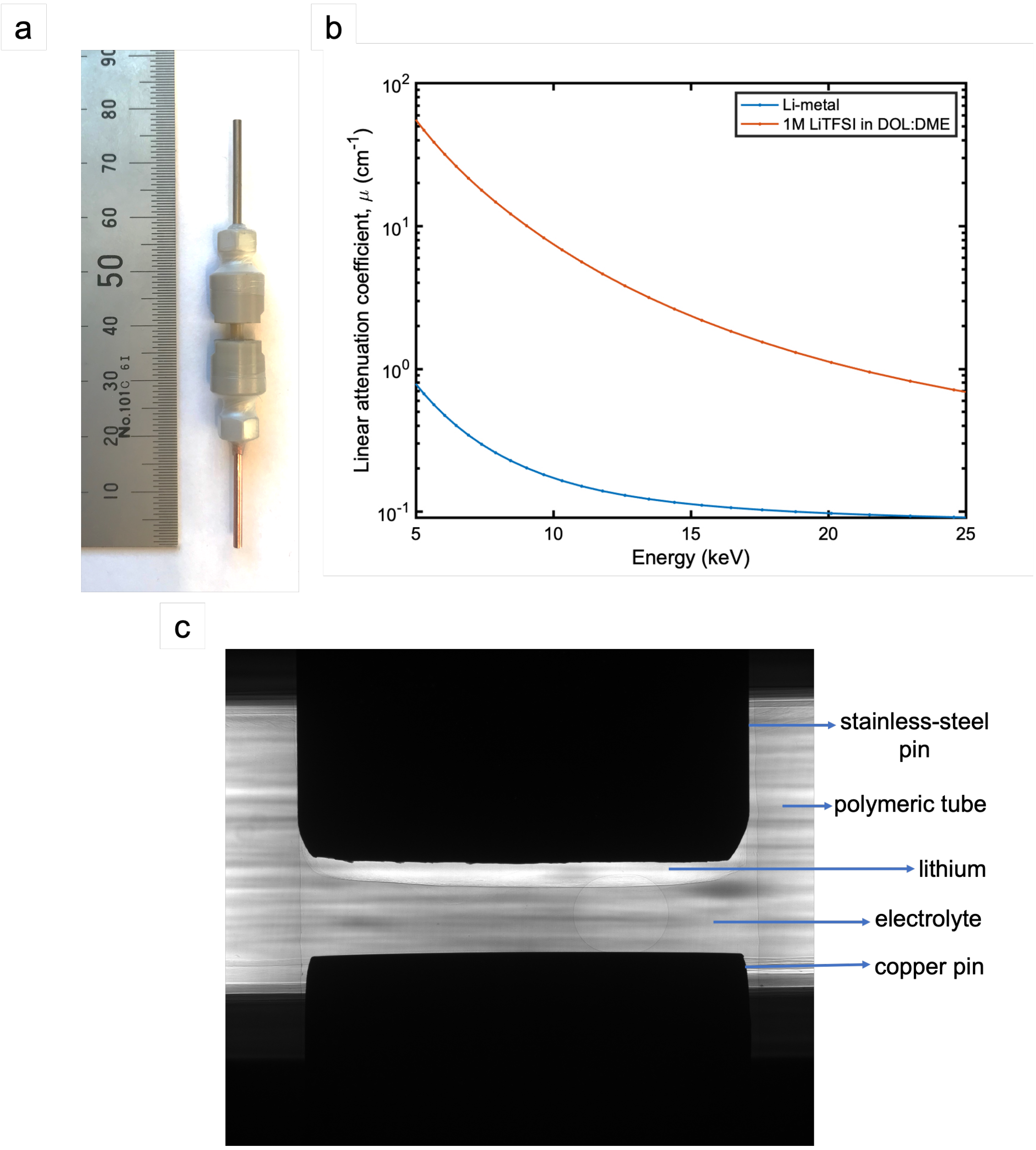

Supplementary Figure 1. Experimental setting for operando X-ray tomographic microscopy. a, Optical image of operando electrochemical cell. **b,** Calculated X-ray attenuation coefficients of Li metal and electrolyte used in our experiment. The linear attenuation coefficient was calculated based on the electrolyte composition and density with the help of NIST X-Ray Form Factor, Attenuation, and Scattering Tables[1]. **c**, Representative projection of the operando cell acquired at low resolution.

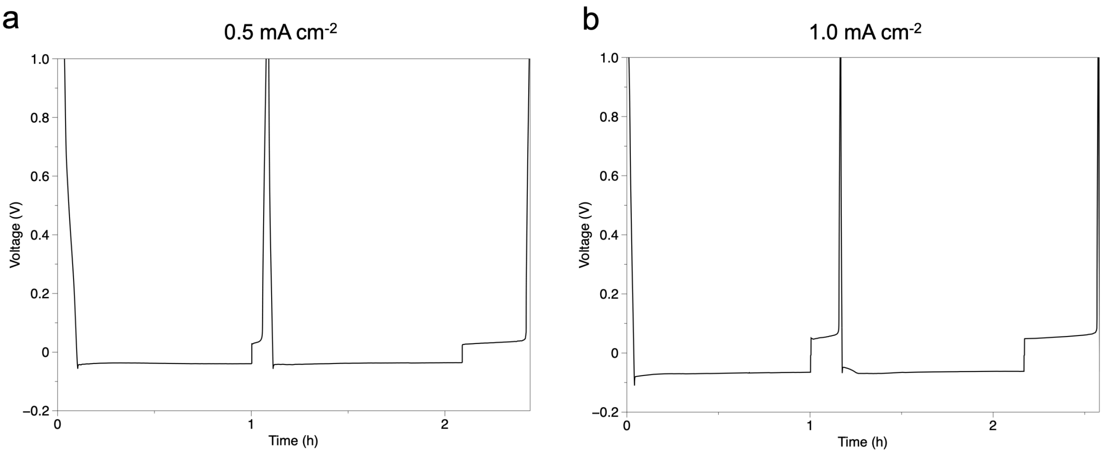

Supplementary Figure 2. **Voltage profiles of consecutive plating/stripping cycles in operando cell. a,** at 0.5 mA cm^{-2}. **b,** at 1.0 mA cm^{-2}. The testing temperature is 20 °C and the cell configuration is Li||Cu cell.

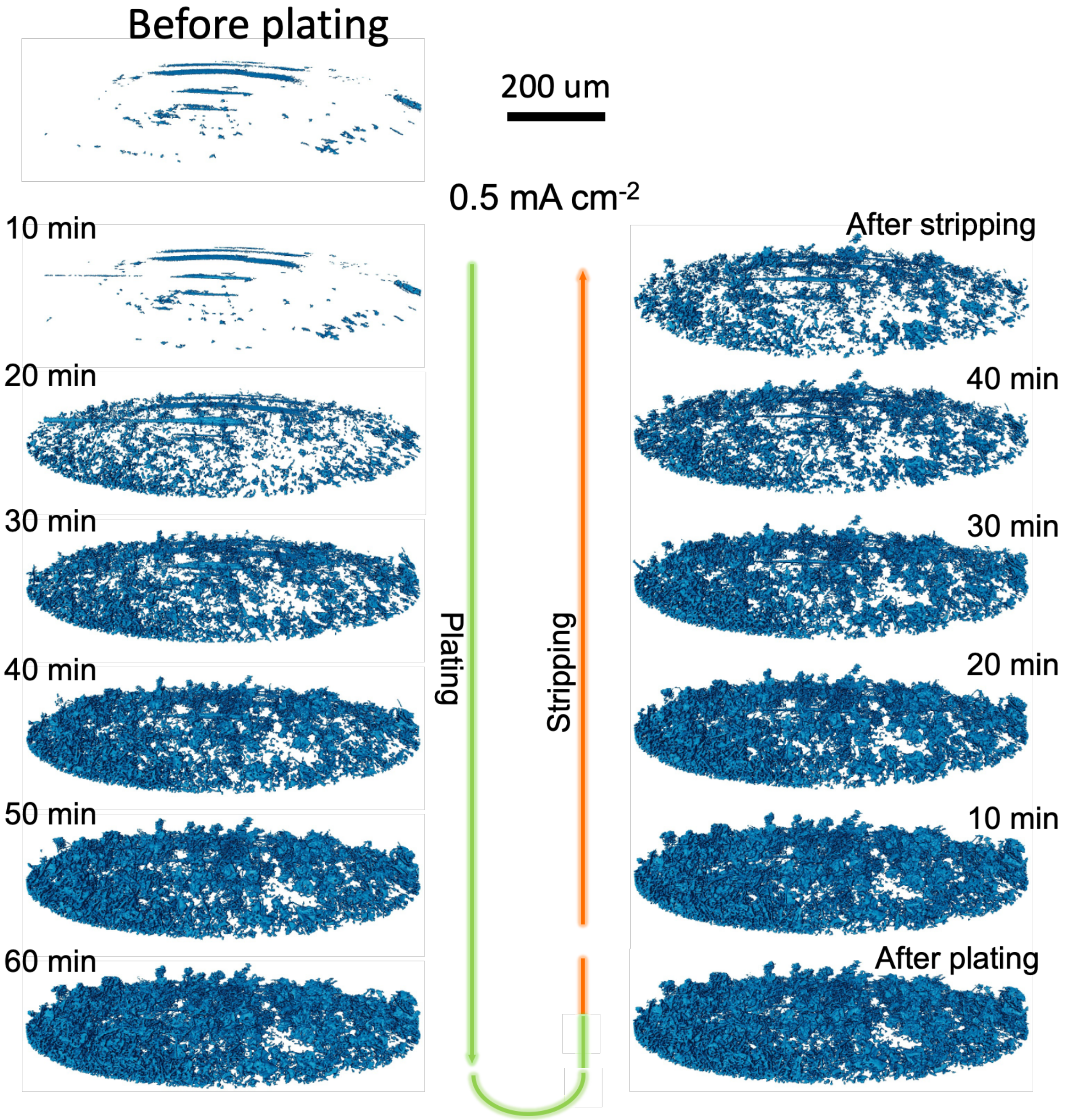

Supplementary Figure 3. 3D renderings of segmented Li during plating/stripping at 0.5 mA cm^{-2}. Tomograms are taken before plating; during plating for 10 min, 20 min, 30 min, 40 min, 50 min, 60 min; after plating; during stripping for 10 min, 20 min, 30 min, 40 min; and after stripping.

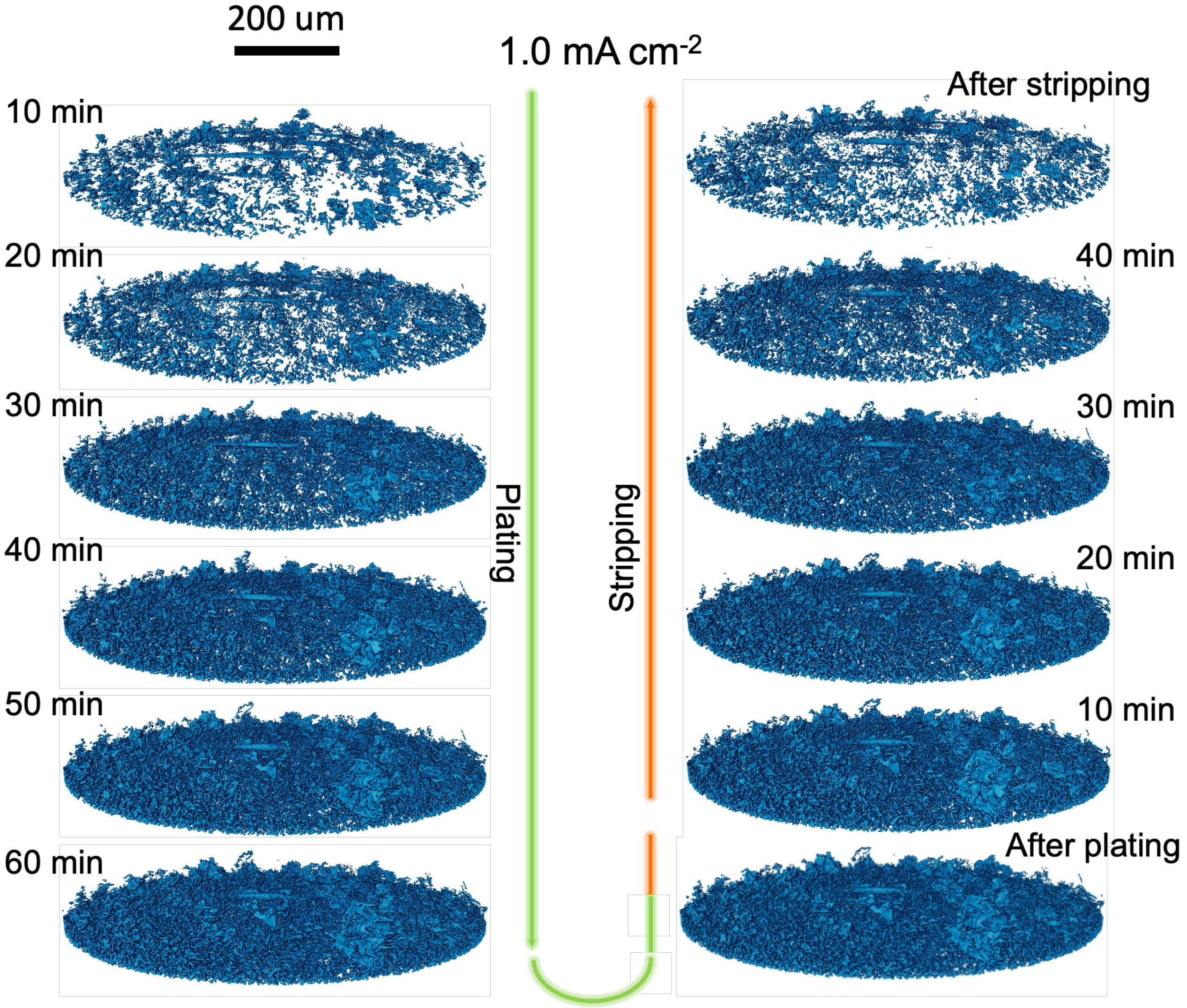

Supplementary Figure 4. 3D renderings of segmented Li during plating/stripping at 1.0 mA cm^{-2}. Tomograms are taken before plating; during plating for 10 min, 20 min, 30 min, 40 min, 50 min, 60 min; after plating; during stripping for 10 min, 20 min, 30 min, 40 min; and after stripping.

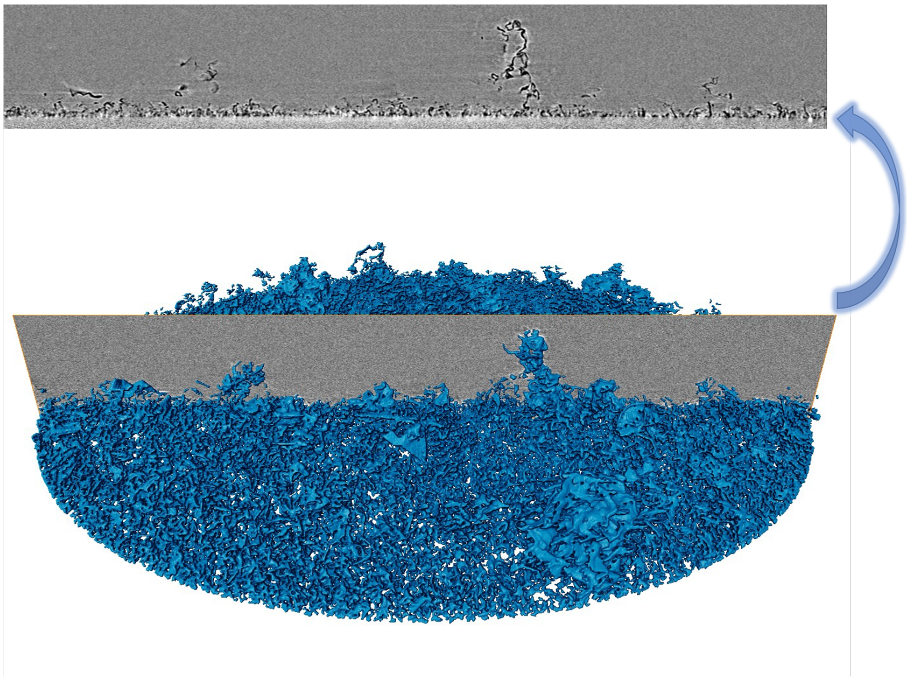

Supplementary Figure 5. Schematic diagram for the extraction of vertical slices from reconstructed 3D tomographic datasets.

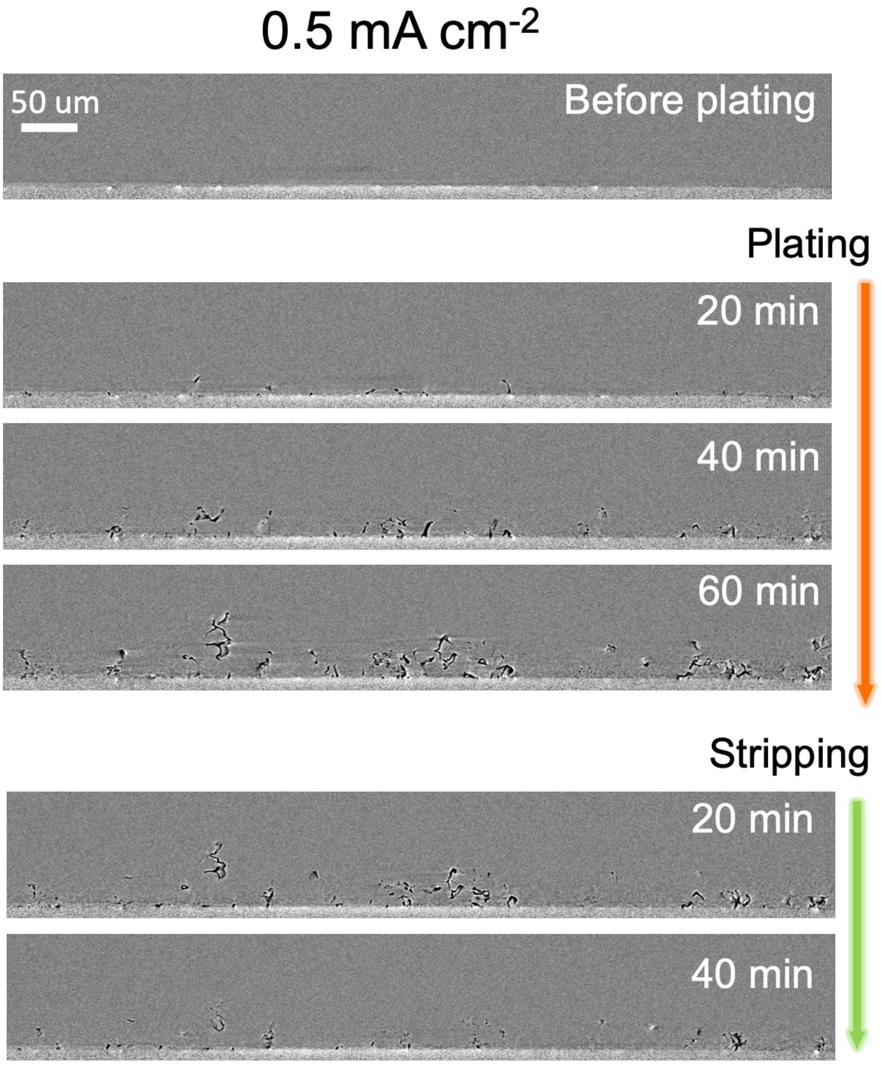

Supplementary Figure 6. Reconstructed cross-sectional slices from cycling at 0.5 mA cm^{-2}. Slices taken before plating; during plating for 20, 40 and 60 min; during stripping for 20 and 40 min.

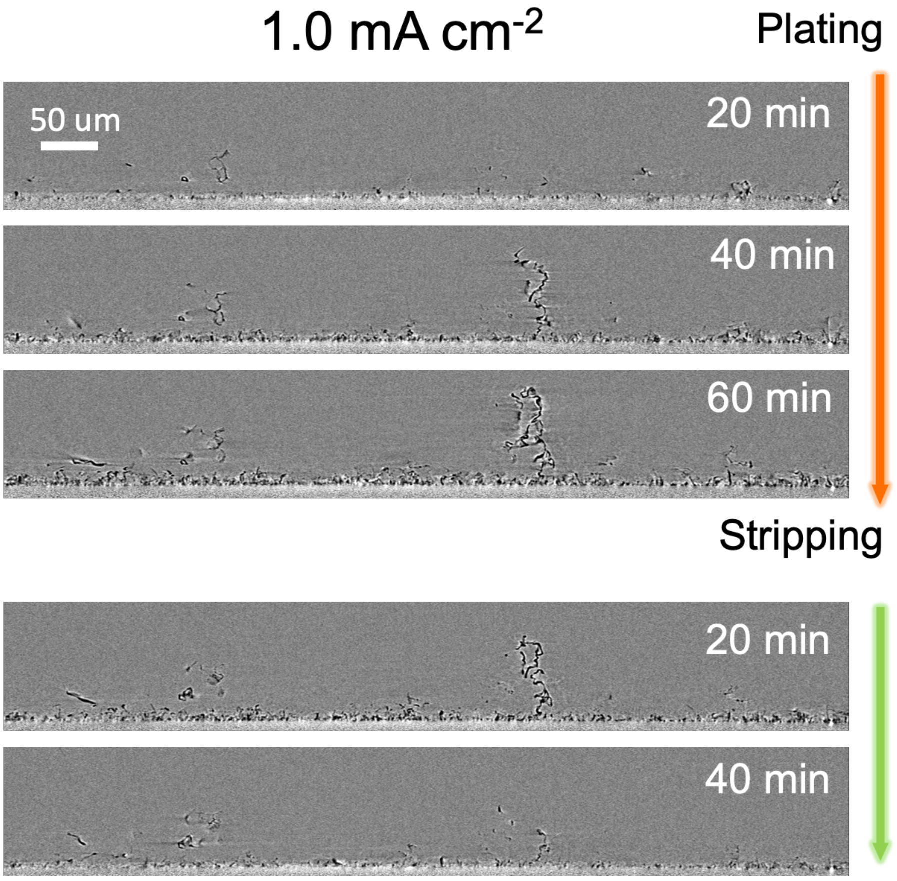

Supplementary Figure 7. Reconstructed cross-sectional slices from cycling at 1.0 mA cm^{-2}. Slices taken during plating for 20, 40 and 60 min; during stripping for 20 and 40 min

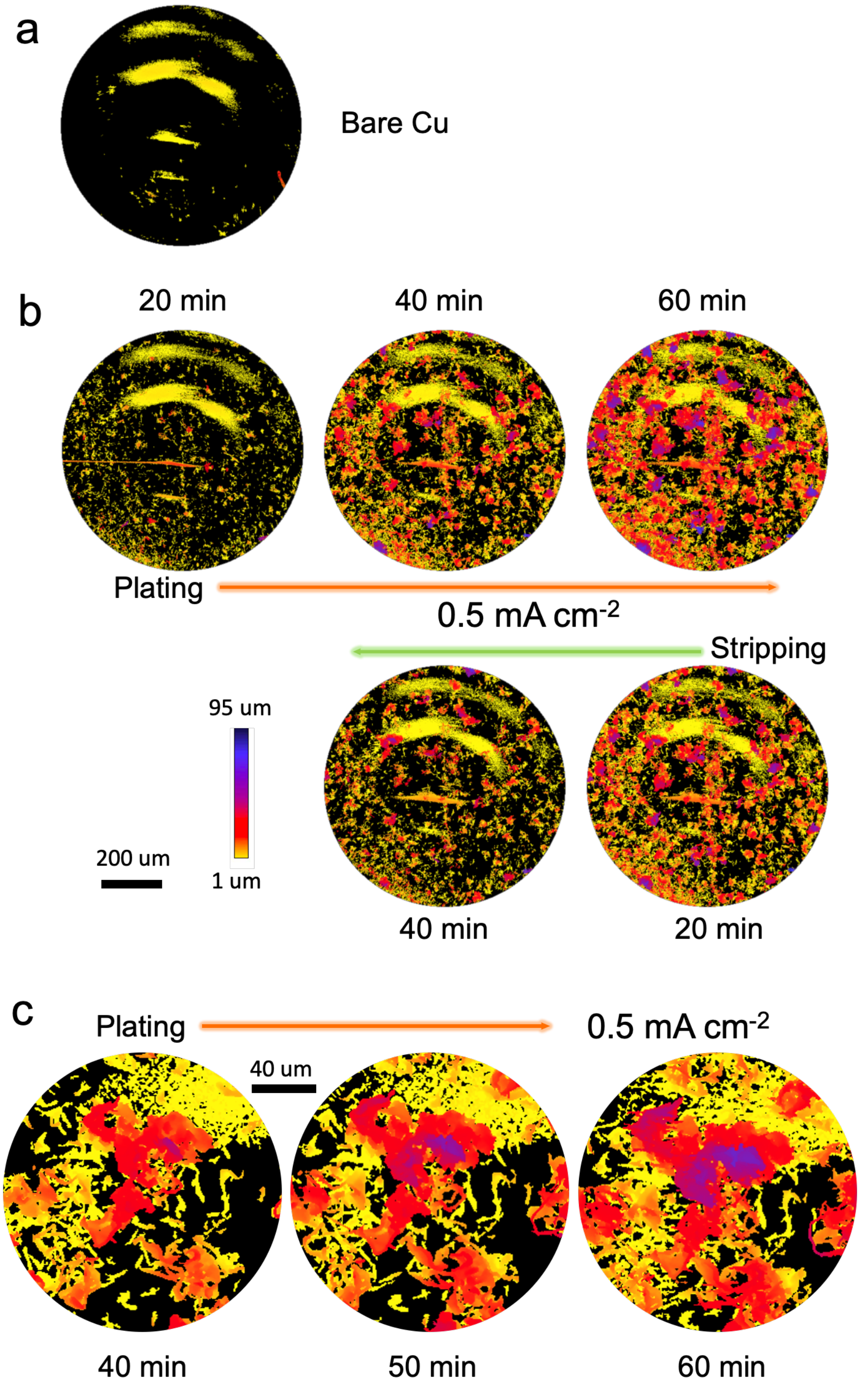

Supplementary Figure 8. Height maps of deposited Li at 0.5 mA cm^{-2}. a, Height map of bare Cu substrate. **b,** Height maps extracted from tomograms taken during plating for 20, 40 and 60 min; during the stripping for 20 and 40 min. **c,** Close up of Li microstructures marked as (i) in Figure 5a during plating at 0.5 mA cm^{-2}.

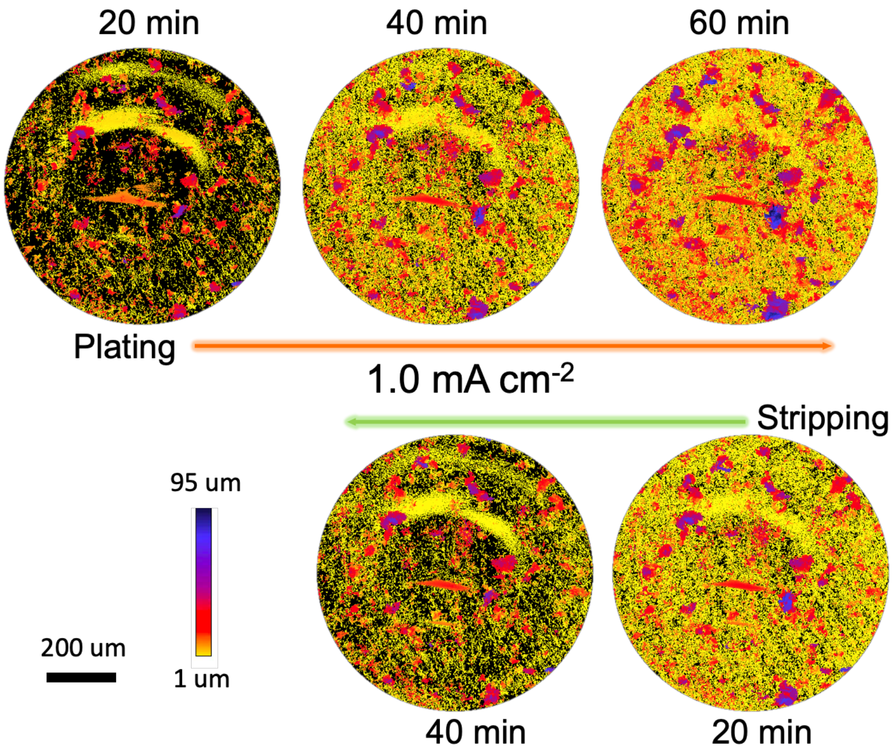

Supplementary Figure 9. Height maps of deposited Li at 1.0 mA cm^{-2}. Height maps extracted from tomograms taken during plating for 20, 40 and 60 min; during stripping for 20 and 40 min.

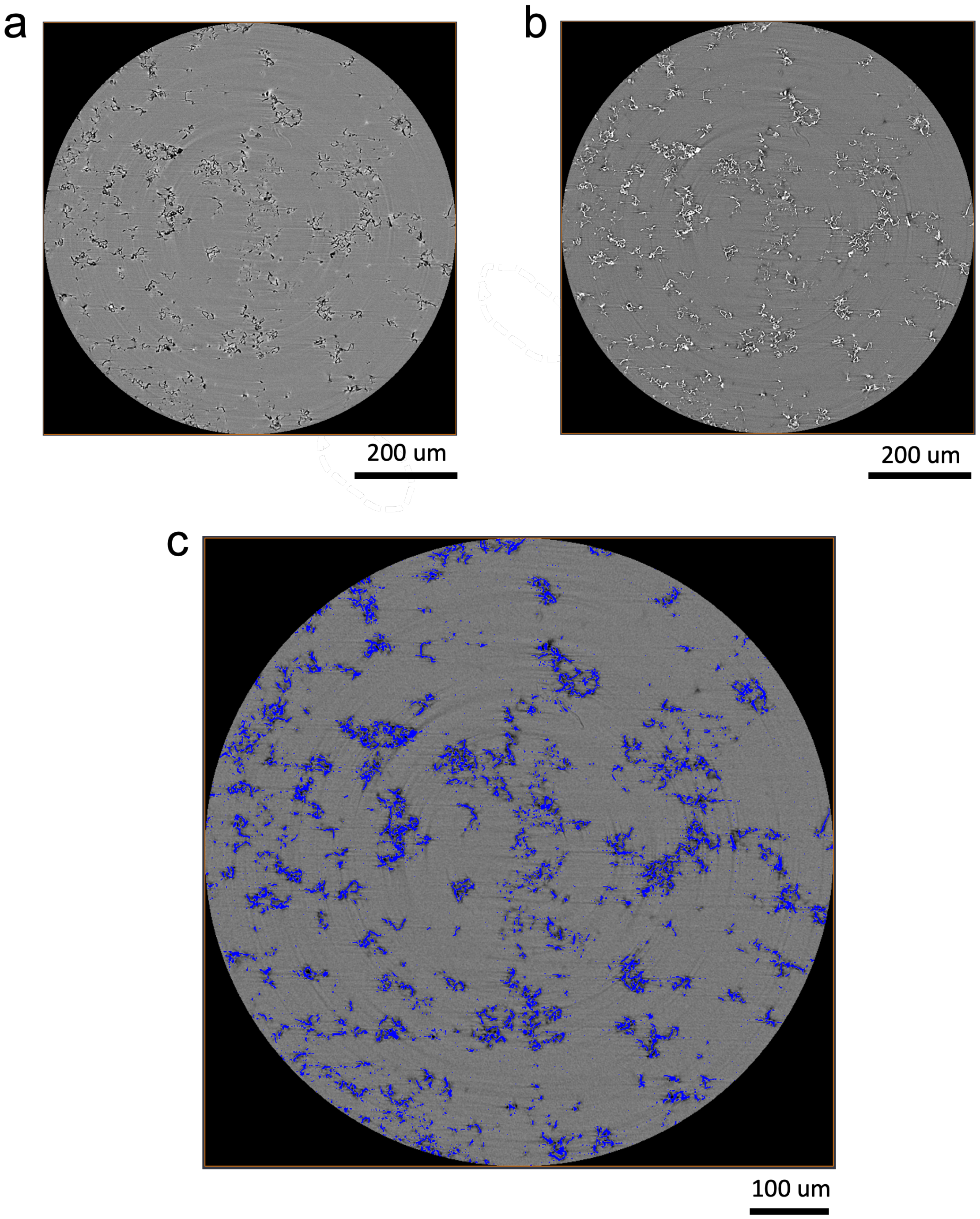

Supplementary Figure 10. Segmentation of Li from liquid electrolyte. a, An axial image slice of a tomogram taken after plating at 0.5 mA cm^{-2} for 1 hour. Distance between this slice and the plane of Cu substrate is 3L=29.25μm. Features with dark contrast are Li, whereas gray region is liquid electrolyte. **b,** Inverted contrast image of slice in **a**. **c,** Segmentation of Li in blue.

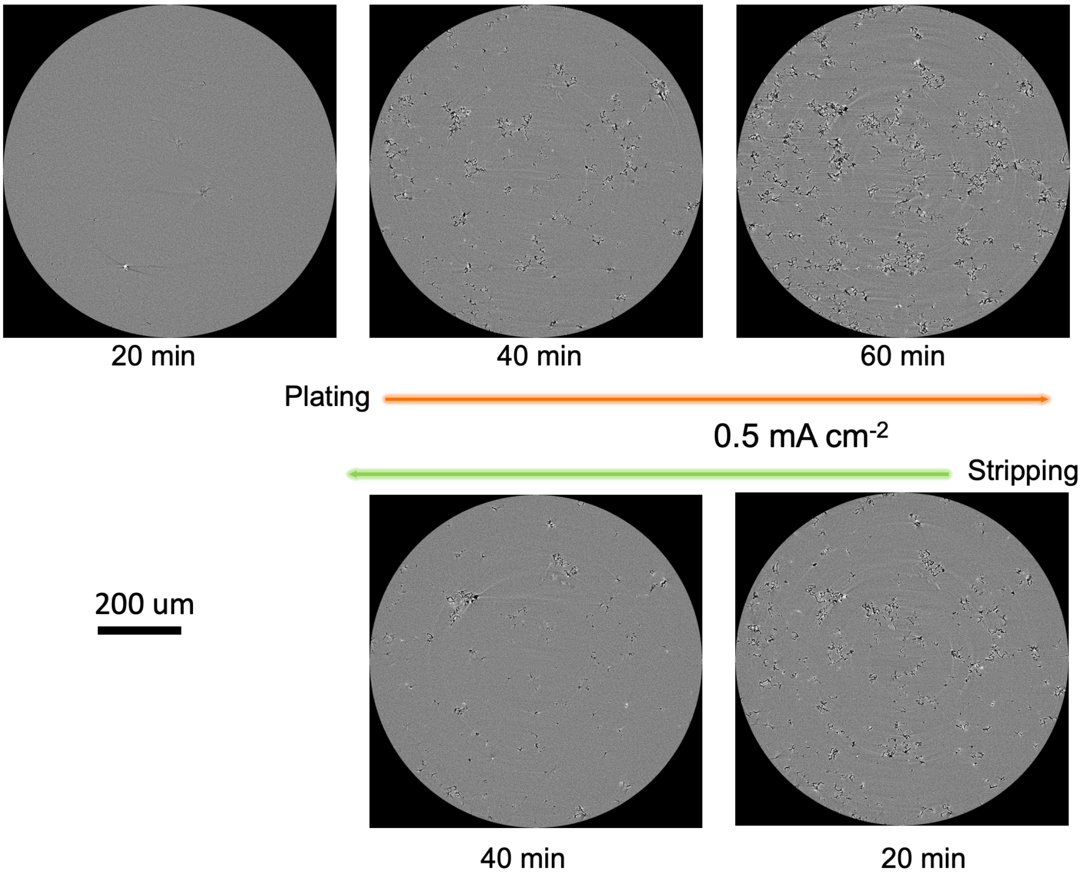

Supplementary Figure 11. Extraction of axial image slices. Slices are extracted from the tomogram taken at 0.5 mA cm^{-2}, during the plating process for 20 min, 40 min and 60 min; during the stripping process for 20 min, 40 min. Distance from the plane of Cu substrate is 3L= 29.25μm.

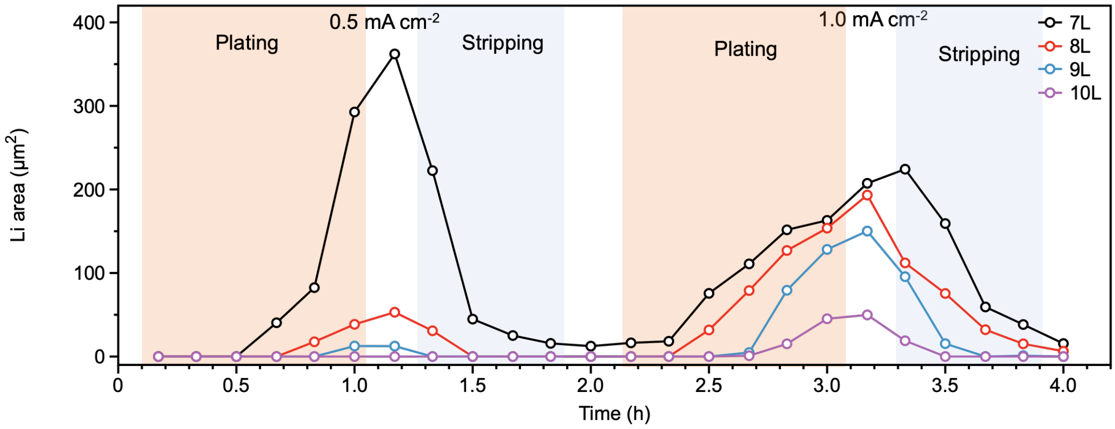

Supplementary Figure 12. Area of deposited Li at different distances from the electrode. Distance is at 7-10 L (1L=9.75 μm) from the Cu surface and the plating/stripping current is 0.5 mA cm^{-2}. The white areas between colored areas are corresponding to the tomograms taken after plating or stripping process.

Supplementary Note 1: Method for Segmentation of Li phase

Segmentation of Li phases in Figure 2, 4 and 6 are carried out by using the following procedures:

Step 1 Loading dataset

As shown in supplementary Figure 13, the entire volume was loaded into in Avizo software, with a defined pixel size of 0.325 µm (detector pixel size = 6.5 µm, objective = x20, pixel size = (6.5 µm / x20) = 0.325 µm). Due to the processing method, the image here is inverted and does not represent the original orientation of the sample. In the Operando measurement, the Li metal electrode is on top, and the copper electrode is in the bottom . Therefore, the top region in this image, which appears as light grey, is the copper electrode pin. The black region with greyscale value of 0 is the Li that has been deposited. The bottom region, which appears as darker grey, is the electrolyte.

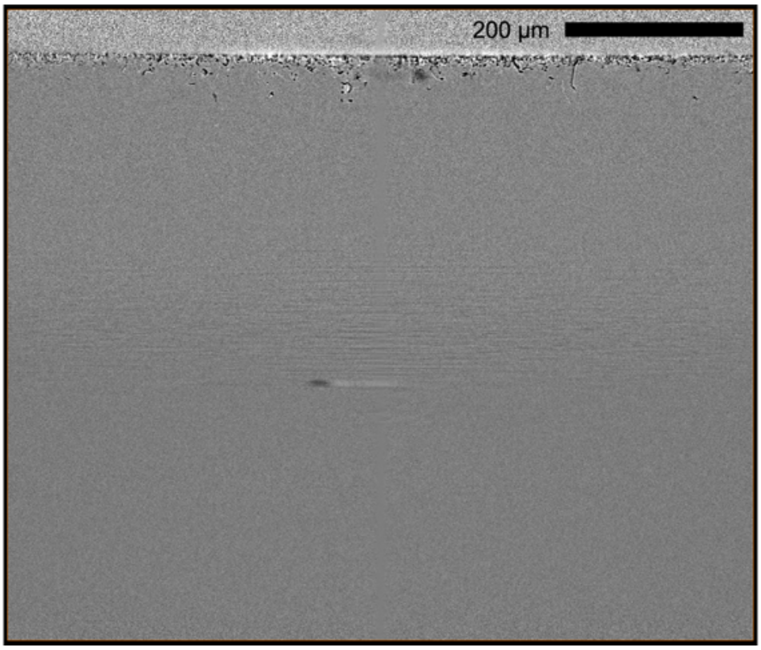

Supplementary Figure 13. Example slice taken from the reconstructed 3D tomogram after plating at 1.0 mA cm^{-2}. The number of pixels is 1280 and it is viewed in the YZ slice,

Step 2 Inversion of the greyscale colourmap

This step is done for next cropping steps since the volume needs to be cropped before the filter is applied to save computational cost. As shown in Supplementary Figure 14, the regions outside the area of interest will be given a greyscale value of 0 (black) before the volume is cropped.

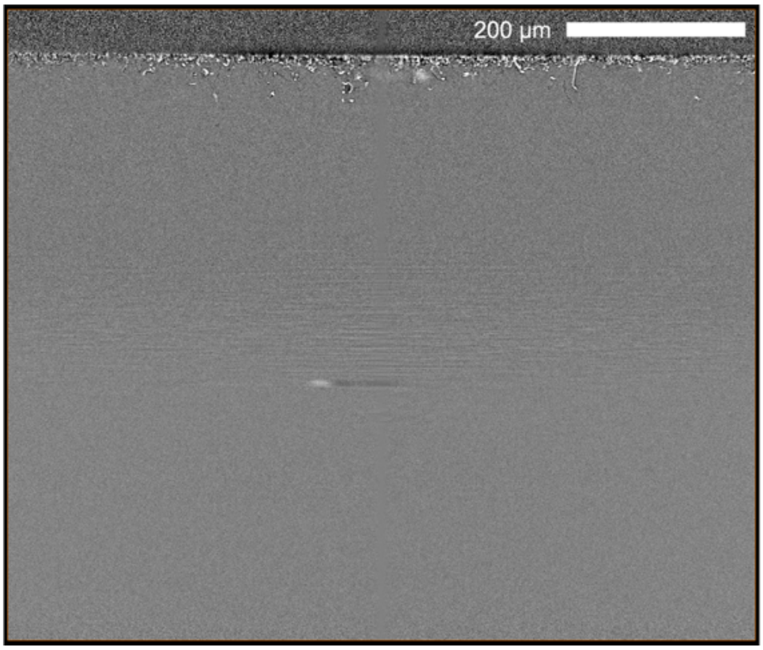

Supplementary Figure 14. Example slice with the greyscale map inverted. It is viewed in the YZ slice.

Step 3 Cropping the volume

The volume is cropped by using a box to keep slices of interest, see Supplementary Figure 15. The volume after cropping is shown in Supplementary Figure 16 where the copper electrode and the region of Li is kept.

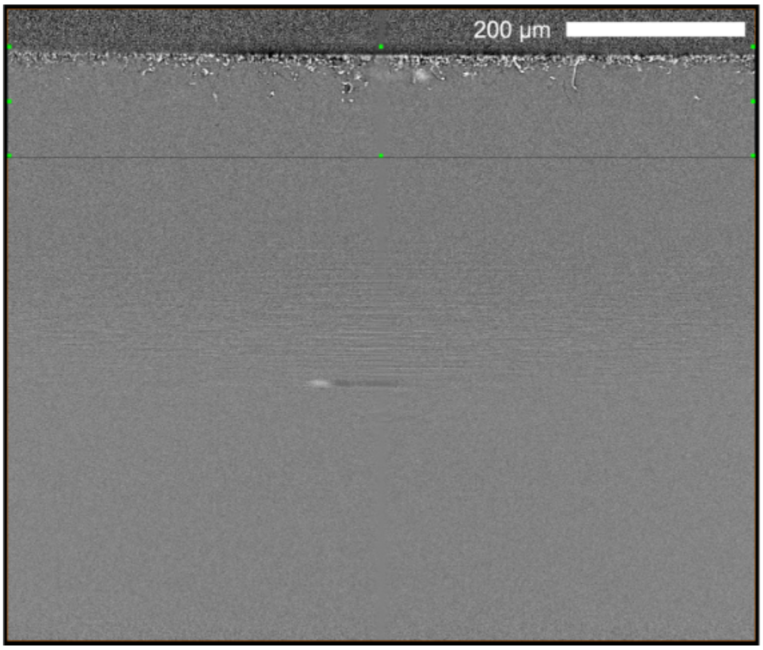

Supplementary Figure 15. Masking of the volume in the z-axis. The z-axis is along the axis of rotation. This image is viewed in the YZ slice.

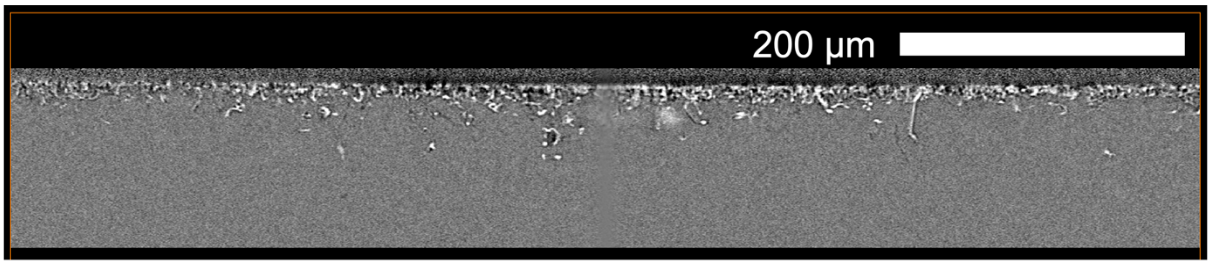

Supplementary Figure 16. The volume after the cropping. It is viewed in the YZ slice.

Step 4 Applying filter

As shown in Supplementary Figure 17, a median filter is applied to remove salt and pepper noise which would later affect the segmentation process.

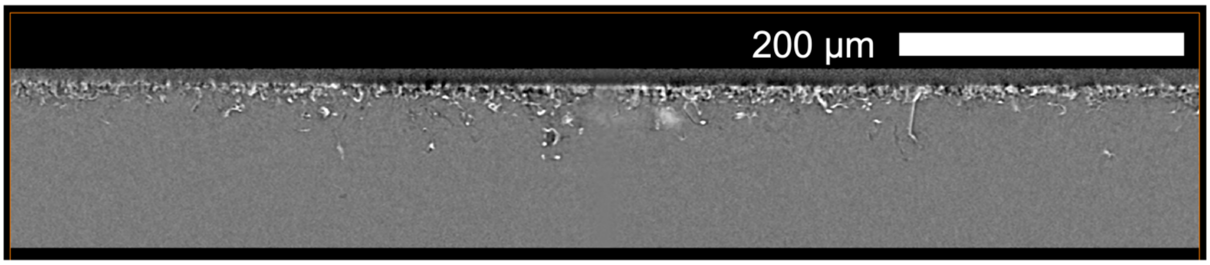

Supplementary Figure 17. Example slice after a median filter was applied. It is viewed in the YZ slice.

Step 5 Cropping for horizontal slices

The horizontal slices Figure 6a and Supplementary Figure 11 are cropped after applying filter. An example of masking is shown in the XY plane (Supplementary Figure 18). The purpose of this masking is to remove regions that are not reconstructed with all angles and to preserve the region of interest only.

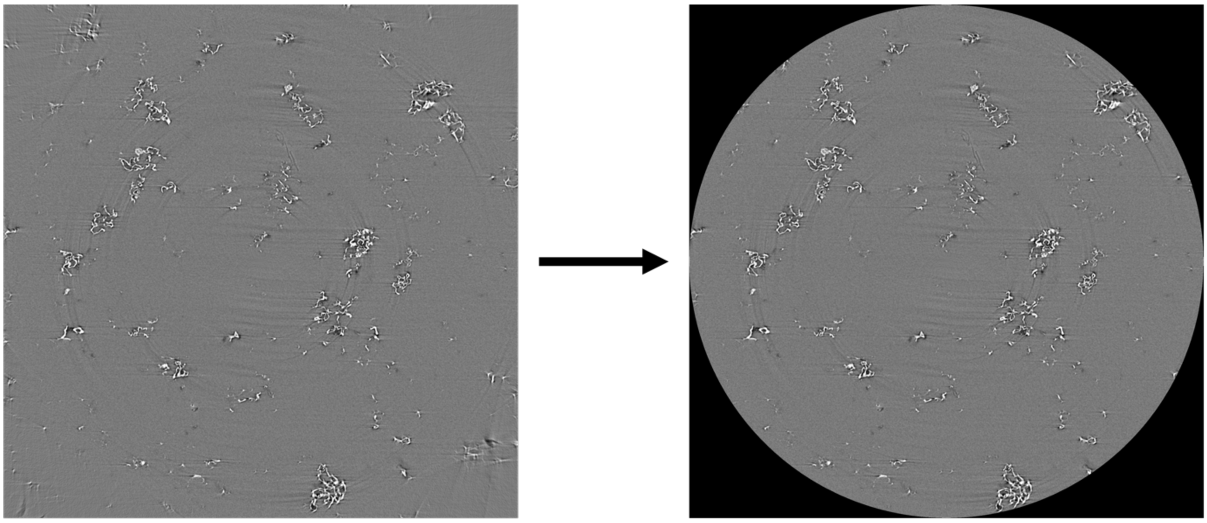

Supplementary Figure 18. Example horizontal slices after masking. It is viewed in the XY slice.

Step 6 Binarization and Visualization

The volume was binarized by using the ‘Interactive Thresholding’ tool in Avizo (Supplementary Figure 10,19). The lower limit for thresholding was set to 37500, and the upper limit was set to the maximum greyscale value which is enabled due to the earlier greyscale inversion.

The binarized pixels is segmented from the surrounding pixels (Supplementary Figure 20) and reconstructed to obtain 3D tomograms for the visualization of Li microstructures (Supplementary Figure 21).

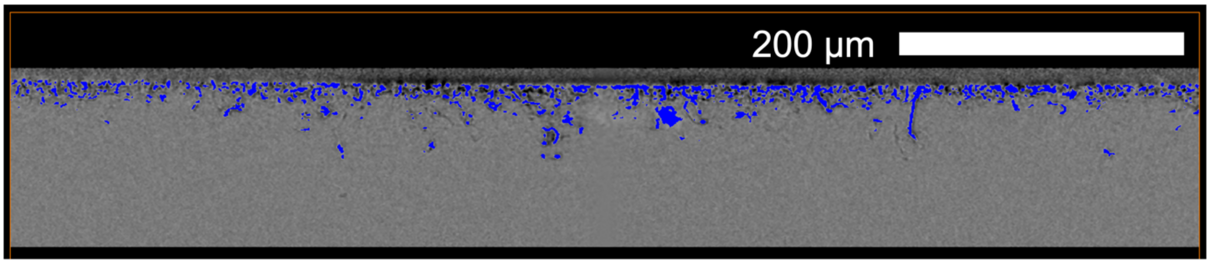

Supplementary Figure 19. Example slice showing a mask on pixels with the threshold. It is viewed in the YZ slice.

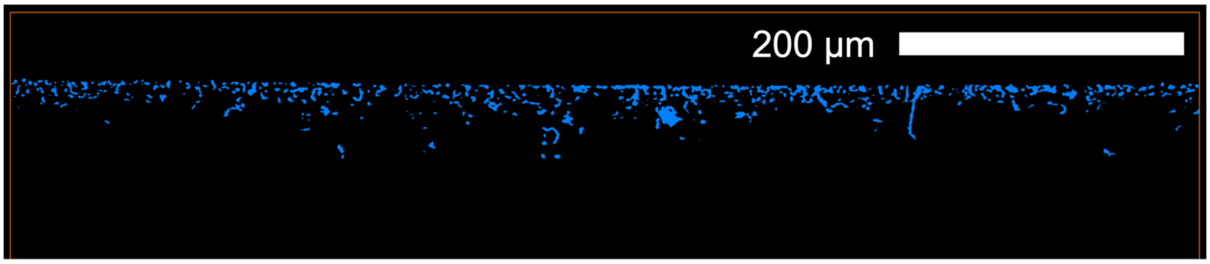

Supplementary Figure 20. Example slice showing the binarized pixels. The value of black pixel is 0 and that of blue pixel is 1. It is viewed in the YZ slice.

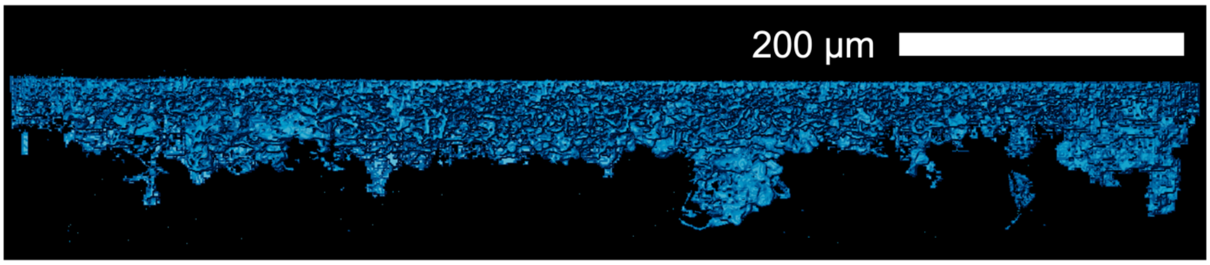

Supplementary Figure 21. Visualization of the binarized volume. It is viewed in the YZ slice.

Supplementary reference

1 Chantler, C. *et al.* X-Ray Form Factor, Attenuation and Scattering Tables (version 2.1).[Online] Available: http://physics. nist. gov/ffast [Monday, 29-Jul-2013 23: 22: 42 EDT]. National Institute of Standards and Technology, Gaithersburg, MD. Originally published as Chantler CT. *J. Phys. Chem. Ref. Data* **29**, 597-1048, (2000).
