# Supplementary material for: Investigating microstructure evolution of lithium metal during plating and stripping via operando X-ray tomographic microscopy
Source: Nat Commun. 2023 Feb 15;14:854. doi: 10.1038/s41467-023-36568-z (PMC9931753; doi:10.1038/s41467-023-36568-z)
Supplement: Supplementary file 2 — Peer Review File [file 41467_2023_36568_MOESM2_ESM.pdf]

nature portfolio

# Peer Review File

## Investigating microstructure evolution of lithium metal during plating and stripping via operando X-ray tomographic microscopy

**Open Access** This file is licensed under a Creative Commons Attribution 4.0 International License, which permits use, sharing, adaptation, distribution and reproduction in any medium or format, as long as you give appropriate credit to the original author(s) and the source, provide a link to the Creative Commons license, and indicate if changes were made. In the cases where the authors are anonymous, such as is the case for the reports of anonymous peer reviewers, author attribution should be to 'Anonymous Referee' followed by a clear attribution to the source work. The images or other third party material in this file are included in the article's Creative Commons license, unless indicated otherwise in a credit line to the material. If material is not included in the article's Creative Commons license and your intended use is not permitted by statutory regulation or exceeds the permitted use, you will need to obtain permission directly from the copyright holder. To view a copy of this license, visit http://creativecommons.org/licenses/by/4.0/.

REVIEWER COMMENTS

Reviewer #1 (Remarks to the Author):

The authors report a very interesting operando X-ray tomography study of Li dendrite formation in an operating battery. They successfully track the deposition and dissolution dynamics of Li over two charge/discharge cycles with different current densities. The data quality and some of the analysis are quite impressive. Specifically, the contrast between Li and other materials (for example Cu or the electrolyte) is clear. The spatial resolution is sufficient to distinguish small branches of the Li needle. The formation of mossy Li is quite clear in the height maps. Although there are previous reports of tomography of operando Li batteries (such as, DOI: 10.1021/acsnano.6b03939 or DOI: 10.1021/acs.jpcc.7b01414), this work is by far the highest quality and demonstrates the potential of the x-ray tomography to make valuable contributions to Li battery science. That being said, the battery science portion of the manuscript would be significantly strengthened by collecting and analyzing a carefully constructed, systematic data set. One or two cycles, at differing current densities, on the same sample is not sufficient to understand the detailed, history-dependent processes involved.

Specific questions for the authors:

1. Tomograms were taken every 10 minutes. But the manuscript does not indicate how long it takes to obtain a tomographic data set. Since the Li dendrite keeps growing during the data collection, it is important that the time to obtain a tomogram is short relative to the characteristic time scale of dendrite formation. The authors need to demonstrate that they are working in a reasonable time range.

2. The authors compared the morphology between 1st cycle and 2nd cycle. The authors then attribute the difference in morphology and voltage profile to the differing current densities. However, these data are insufficient to make this claim, as there are many variables that differ between the 1st and 2nd cycles (for example, the residual of SEI or Li dendrites after the 1st cycle, changes in the number of nucleation sites, etc.). I recommend that the authors conduct two experiments that run several cycles without changing the current density (one at 0.5 mA/cm2, the other at 1.0 mA/cm2) on separate samples.

3. The voltage profile of Figure 2b: the lower overpotential at high current density may not be due to the current density. It may also be caused by the formation of some new nucleation sites or a change in the SEI layer after the 1st cycle or a decrease in the fresh exposed Cu surface in the 2nd cycle. Again, more data could sort this out.

4. One question about Figure 2d and e: the last picture from the 1st cycle does not look similar to the 1st figure of the 2nd cycle. The inactive Li in Figure 2d (after stripping) is not seen in Figure 2e. If the images are not from the same spot, that needs to be mentioned. Additionally, I recommend the authors add a 3d rendering volume of Li dendrite in Figure 2 and mark the vertical slice where Figure 2d and2e were taken.

5. In page 7, the observation of inactive Li: “caused by the encapsulation by the electronically insulating SEI…”. The experimental evidence of inactive Li is clear, but the reason for the formation of inactive Li (due to SEI or not) is not supported by the data.

6. The authors propose in Fig. 3C that some Li becomes inactive during plating. Again, this may or may not be true - the evidence is not clear. This Li may become inactive quickly when stripping, but not during plating. If inactive Li is formed during plating, the evidence should be that Li dendrite stops growing at some point of time during the plating. More data is needed.

7. The part about horizontal slices and the analysis of Li area at different heights is impressive. But it is important to add an explanation in the methods section. How to calculate mathematically the Li area, what is the standard to determine the isolated region of Li in Figure 5, how the purple region in Figure 6 is defined mathematically.

8. Page 5, 10th line of the last paragraph: “It is worth noting that the overpotential for nucleation of Li at high current density (1.0 mA cm-1) is lower.” The unit should be 1.0 mA cm-2. Same errors happen in the caption of Fig. 2.

9. The current densities in these studies are small relative to those of practical batteries. I assume these current densities were chosen to slow down the growth of the dendrites so that it is possible to obtain tomograms. Since we already know that current density affects dendrite formation, eventually we need to study batteries under realistic operating conditions. Can x-ray tomography access this range of parameter space?

Reviewer #2 (Remarks to the Author):

review attached

Reviewer #3 (Remarks to the Author):

- What are the noteworthy results?

Authors visualized microstructure evolution of lithium plating as a function of time and current density, distinguishing different types of structures and distinguishing the formation of inactive lithium.

- Will the work be of significance to the field and related fields? How does it compare to the established literature? If the work is not original, please provide relevant references.

This work is significant and will be of interest to many research groups. I agree that it is challenging to do operando experiments with sufficient resolution. However authours need to provide better explanation of novelty of their approach. In the introduction authors stated that it is challenging to visualize and provided references of past work that used heavier elements, formation of cracks or with respect to a separator, claiming there was no direct and continuous tracking of the evolution of Li. What is different in the approach presented here that enabled this team to do what others could not? This is not clear, especially for readers not familiar with the XTM. Authors should better highlight similarities and differences in the approaches and findings between current work and previous reports, and should also should include other citations and their discussion, for example work conducted at the Advanced Photon Source by Seitzman featuring solid electrolyte.

Seitzman, N.; Bird, O. F.; Andrykowski, R.; Robbins, S.; Al-Jassim, M. M.; Pylypenko, S., Operando X-ray Tomography Imaging of Solid-State Electrolyte Response to Li Evolution under Realistic Operating Conditions. ACS Applied Energy Materials 2021, 4 (2), 1346-1355.

- Does the work support the conclusions and claims, or is additional evidence needed?

This paper is written well, and all conclusions are supported by the data. Figure 2 needs additional markers and its discussion should be revised to make it clear what area in Fig 2 is being discussed. Fig. 2 shows several regions of interest- one area was highlighted in yellow circle, another in red oval, and third was marked with green rectangle. In some places authors referred to specific area and how it was marked, but in some places, they referred to area without indicated how it was marked (for example green area). Also, there was additional area of interest discussed, with mossy and needle-like structure, but the area is not marked in the Fig 2.

- Are there any flaws in the data analysis, interpretation and conclusions? - Do these prohibit publication or require revision?

Authors should make a stronger statement of the impact of this study on design of ideal structures.

- Is the methodology sound? Does the work meet the expected standards in your field?

Yes

- Is there enough detail provided in the methods for the work to be reproduced?

Yes

## A Point-by-Point Response to Reviewers (NCOMMS-22-17503-T)

***Reviewer #1***

*The authors report a very interesting operando X-ray tomography study of Li dendrite formation in an operating battery. They successfully track the deposition and dissolution dynamics of Li over two charge/discharge cycles with different current densities. The data quality and some of the analysis are quite impressive. Specifically, the contrast between Li and other materials (for example Cu or the electrolyte) is clear. The spatial resolution is sufficient to distinguish small branches of the Li needle. The formation of mossy Li is quite clear in the height maps. Although there are previous reports of tomography of operando Li batteries (such as, DOI: 10.1021/acsnano.6b03939 or DOI: 10.1021/acs.jpcc.7b01414), this work is by far the highest quality and demonstrates the potential of the x-ray tomography to make valuable contributions to Li battery science. That being said, the battery science portion of the manuscript would be significantly strengthened by collecting and analyzing a carefully constructed, systematic data set. One or two cycles, at differing current densities, on the same sample is not sufficient to understand the detailed, history-dependent processes involved.*

**Authors' response:** We thanks for the positive statements about our work and the mentioned references. We have added these in the revised manuscript (Ref. 40 and Ref. 41) to better contrast our work to previous results.

*Specific questions for the authors:*

*1. Tomograms were taken every 10 minutes. But the manuscript does not indicate how long it takes to obtain a tomographic data set. Since the Li dendrite keeps growing during the data collection, it is important that the time to obtain a tomogram is short relative to the characteristic time scale of dendrite formation. The authors need to demonstrate that they are working in a reasonable time range.*

**Authors' response:** The acquisition time per tomogram is 63s, i.e. short with respect to the deposition process. More specifically, 1000 projections were taken for the acquisition of each tomogram and the exposure time for each projection is 50 ms. In addition, there is an overhead time of 13ms per each projection and thus the total time for taking one tomogram is 63000 ms, that is, 63s. Details on the acquisition time was added in the methods section of the revised manuscript on page 18 as well as in the introduction on page 4.

*2. The authors compared the morphology between 1st cycle and 2nd cycle. The authors then attribute the difference in morphology and voltage profile to the differing current densities. However, these data are insufficient to make this claim, as there are many variables that differ between the 1st and 2nd cycles (for example, the residual of SEI or Li dendrites after the 1st cycle, changes in the number of nucleation sites, etc.). I recommend that the authors conduct two experiments*

*that run several cycles without changing the current density (one at 0.5 mA/cm2, the other at 1.0 mA/cm2) on separate samples.*

**Authors' response:** We fully understand the concern of reviewer and it is the first question we faced when the experiment was designed. Both the cycling history and the current density will influence morphology of deposited Li and ideally one would like to disentangle the two effects. However, to follow the evolution of the microstructure it is central to actually compare the growth and dissolution of structures and quantitatively analyze the changes in exactly the same area/volume (like in Figures 3 and 5 in the revised manuscript). This is not possible if the tomograms are collected from two different cells. With regard to this, we have throughout the revised manuscript clarified that both the second cycle and a higher current density are factors influencing the nucleation and deposition process. In addition, we have added new experimental electrochemical data from two separate operando cells where electrochemical plating/striping was performed at constant current densities (0.5 mAcm$^{-2}$ and 1.0 mA cm$^{-2}$, respectively) for subsequent cycles. The voltage profiles of these two cells are shown in Supplementary Figure 2, see also below. The data shows that the voltage profile in the second cycle at high current density is indeed different from the behavior at lower current density. The two-step profile for nucleation is only observed under high current density, in which the local current density on the area of bare Cu (black area in Figure 5 in revised manuscript) is enhanced since part of Cu surface is covered by inactive Li created in the first cycle. This points to enhanced effects by current density. We have added a discussion on this on page 7 in the revised manuscript.

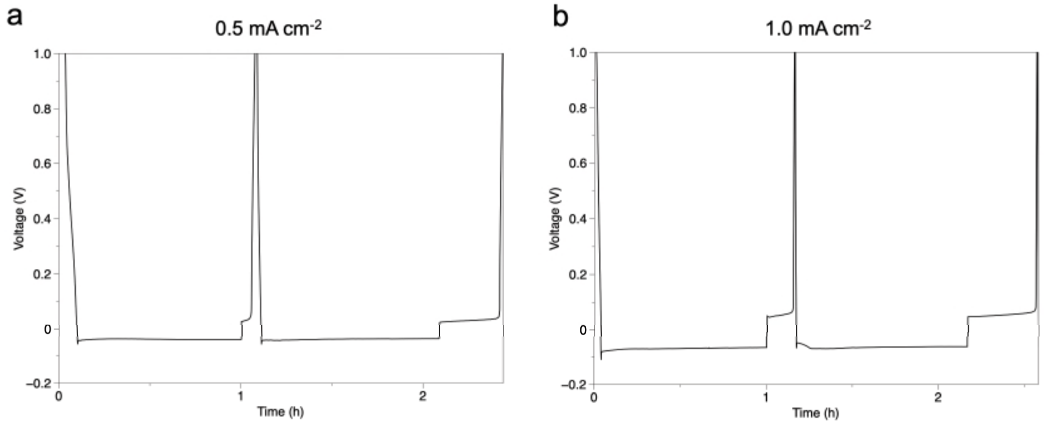


Supplementary Figure 2. Voltage profiles of consecutive plating/stripping cycles in operando cell. a, at 0.5 mA cm$^{-2}$. b, at 1.0 mA cm$^{-2}$.

*3. The voltage profile of Figure 2b: the lower overpotential at high current density may not be due to the current density. It may also be caused by the formation of some new nucleation sites or a change in the SEI layer after the 1st cycle or a decrease in the fresh exposed Cu surface in the 2nd cycle. Again, more data could sort this out.*

**Authors' response:** We agree with the referee that there can also be other factors influencing the nucleation overpotential in the second cycle, as discussed above. From cycling of the operando cell offline (i.e. not during the tomography experiment) we can see that the overpotential at higher current density changes from first to second cycle which points to formation of new nucleation sites or change in SEI is indeed an important factor. We have added this to the discussion on the data in the revised manuscript on page 7 and also added the data with consecutive cycles at constant current density of the operando cell, Supplementary Figure 2 (see below), to underpin this result.

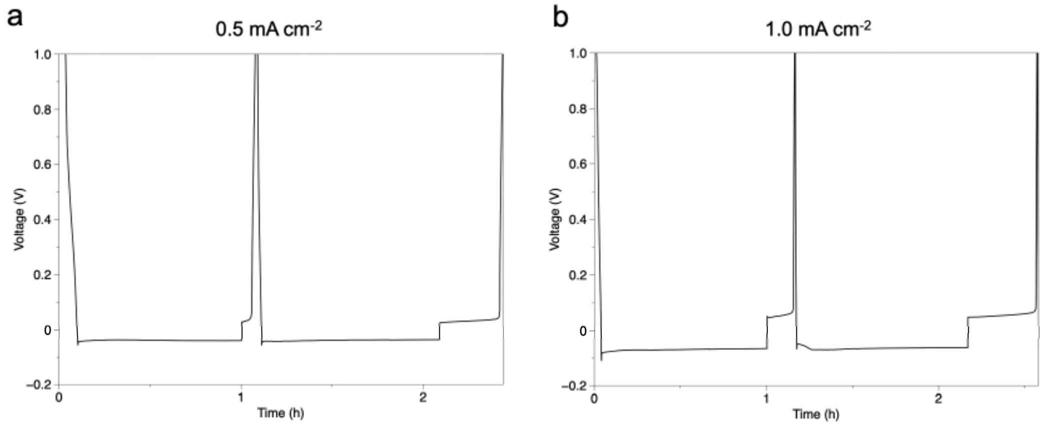


Supplementary Figure 2. Voltage profiles of consecutive plating/stripping cycles in operando cell. a, at 0.5 mA $cm^{-2}$. b, at 1.0 mA $cm^{-2}$.

*4. One question about Figure 2d and e: the last picture from the 1st cycle does not look similar to the 1st figure of the 2nd cycle. The inactive Li in Figure 2d (after stripping) is not seen in Figure 2e. If the images are not from the same spot, that needs to be mentioned. Additionally, I recommend the authors add a 3d rendering volume of Li dendrite in Figure 2 and mark the vertical slice where Figure 2d and2e were taken.*

**Authors' response:** The slices from the first and second cycle were indeed different, and we agree that this can cause confusion. In the revised manuscript, we have adjusted this and now all slices in Figure 3 on page 9 (previously in Figure 2) are taken from the same position in the 3D tomogram. The position is also marked in a 3D rendering of the data added as Supplementary Figure 5, please see also below. We have also clarified that the slices are from the same position in the 3D datasets on page 8.

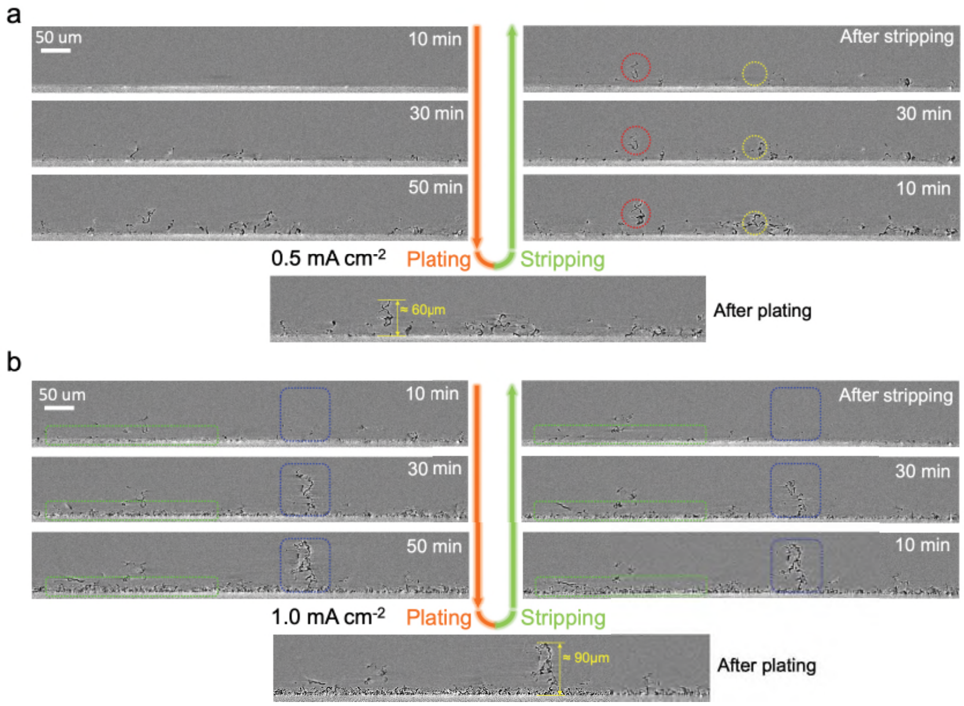


Figure 3 | Operando XTM during plating and stripping at different current densities.

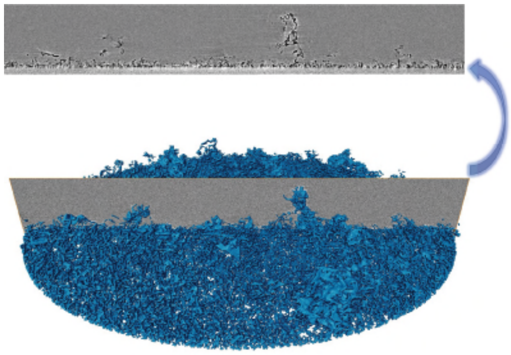

Supplementary Figure 5. Schematic diagram for the extraction of vertical slices from reconstructed 3D tomographic datasets.

*5. In page 7, the observation of inactive Li: "caused by the encapsulation by the electronically insulating SEI…". The experimental evidence of inactive Li is clear, but the reason for the formation of inactive Li (due to SEI or not) is not supported by the data.*

**Authors' response:** We agree with that mechanism for formation of inactive Li is not directly shown in our work. We have on page 9 revised this statement to highlight that we directly observe the formation of inactive Li, and that previous literature suggests the disconnection from the electronically conductive Li network is a major mechanism. Proper references for this are also added.

*6. The authors propose in Fig. 3C that some Li becomes inactive during plating. Again, this may or may not be true - the evidence is not clear. This Li may become inactive quickly when stripping, but not during plating. If inactive Li is formed during plating, the evidence should be that Li dendrite stops growing at some point of time during the plating. More data is needed.*

**Authors' response:** We understand the concern raised by the reviewer. Looking in detail into the heightmaps of Li deposited at 0.5 mA cm$^{-2}$ for 40, 50, 60 minutes and after plating one can see that part of the Li microstructures already exists on the height map after 50 minutes plating and barely shows any change after that during plating, neither during stripping. This suggests that the disconnection of deposited Li from the electronically conductive network may occur at the early stage of stripping, or even during the plating. We have clarified this in the discussion on the data in the revised manuscript on page 11. We have also added the time series of the close-up region, marked with white circle (i) in Figure 5, as Supplementary Figure 8c.

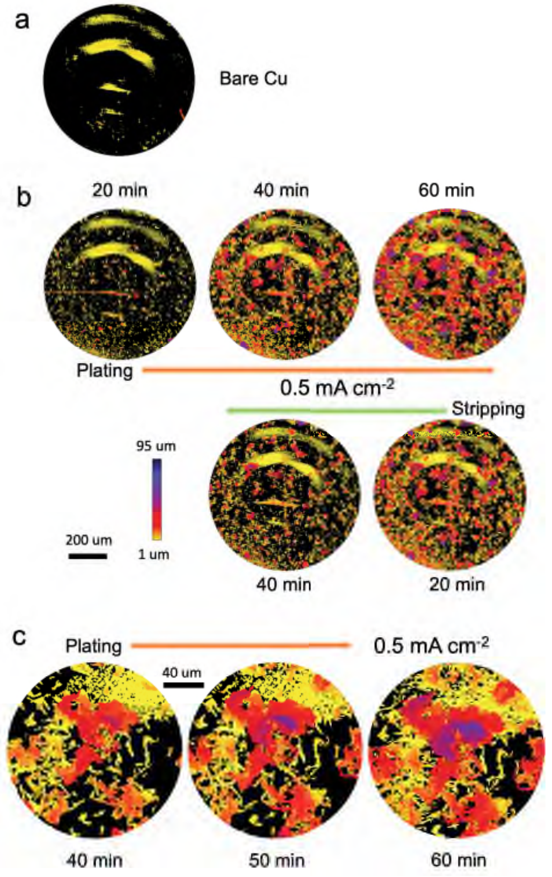


Supplementary Figure 8. Height maps of deposited Li at 0.5 mA cm$^{-2}$. a, Height map of bare Cu substrate. b, Height maps extracted from tomograms taken during plating for 20, 40 and 60 min; during the stripping for 20 and 40 min. c, Close up of Li microstructures marked as cycle (i) in Figure 5a during plating at 0.5 mA cm$^{-2}$.

*7. The part about horizontal slices and the analysis of Li area at different heights is impressive. But it is important to add an explanation in the methods section. How to calculate mathematically the Li area, what is the standard to determine the isolated region of Li in Figure 5, how the purple region in Figure 6 is defined mathematically.*

**<u>Authors' response:</u>** The area of Li in Figure 6 (previous Figure 4) is determined through a segmentation procedure of the black voxels in an individual slice. The number of voxels which are assigned to Li are then multiplied by the surface area of a voxel (0.325µm x 0.325µm) to give the area of Li in a specific slice. A description of this procedure is added to the methods section on page 19.

The isolated regions of Li in Figure 2 (previous Figure 5) were determined by using the Label Analysis module of the AVIZO software package on the segmented Li volumes. Isolated regions are individual segmented Li structures that are not connected to an adjacent region via a segmented voxel. A description of this procedure is added to the methods section on pages 18.

The purple region in Figure 4 (previous Figure 6) is a further segmentation of the reconstructed volume. The same process is followed as for the segmentation of the Li regions but with a different range of greyscale was selected compared to the Li-phase. The above segmentation details are added in the methods section on pages 18.

*8. Page 5, 10th line of the last paragraph: "It is worth noting that the overpotential for nucleation of Li at high current density (1.0 mA cm-1) is lower." The unit should be 1.0 mA cm-2. Same errors happen in the caption of Fig. 2.*

**<u>Authors' response:</u>** The typo was corrected in the revised manuscript, please see in page 9 (caption of previous Figure 2).

*9. The current densities in these studies are small relative to those of practical batteries. I assume these current densities were chosen to slow down the growth of the dendrites so that it is possible to obtain tomograms. Since we already know that current density affects dendrite formation, eventually we need to study batteries under realistic operating conditions. Can x-ray tomography access this range of parameter space?*

**<u>Authors' response:</u>** Studying batteries at higher current densities is indeed highly relevant. We believe that this could be possible with X-ray tomography but would require even faster acquisition times for the full tomogram. In the current experiment with 63s for a full tomogram we could go to slightly higher current densities without blurring the image. Reducing the exposure time per projection and/or reducing the number of projections would be the best option and with current experimental setup one can envisage going to around 30s per tomogram by fully optimizing the conditions and also

the cell design. This could allow experiments with current densities up to 5 mA $cm^{-2}$. With development of synchrotron sources with higher brilliance and detector development (faster detectors) one can of course expect even faster acquisition and accessing processes at higher current density. We have added a comment on this on page 16 in the revised manuscript.

***Reviewer #2*** *:*

*The authors used an operando synchrotron X-ray tomographic microscopy to capture and reveal the formation, growth, and dissolution of electrochemically generated Li microstructures during Li|Cu cell operation. The operando setup allows the authors to record the evolution of deposited Li as a function of time and applied current density (0.5 mA cm-2 and 1 mA cm-2 at 0.5mAh cm-2 and 1 mAh cm-2 capacity for 2 cycles). Data analysis allows the author to distinguish the formation of inactive Li during plating to that formed during stripping. Further data segmentation of Li microstructures shows the structural connectivity of deposited Li at different current densities as well as the formation mechanisms of fractal Li microstructures, which are rapidly growing and fatal to battery safety. Overall, this work has successfully tracked the formation and evolution of electrochemically formed Li microstructures during battery cycling, providing some of new insights into the evolution of Li internal microstructures. Nevertheless, there are some critical issues that needs to be further addressed.*

*1, The Li dendrites presented in the current work attract the reviewer's attention. As we all know, the contrast between Li (either pure Li bulk or electrochemically formed Li microstructures) and the electrolyte is poor. If there were no changes occurring at the Li/liquid electrolyte interface, we may probably have no idea which part is which, as the first panel in Fig. 2d shows. During electrochemical plating, changes occurred at Li/electrolyte interface, as shown in Fig. 2d. The authors assigned the black/dark regions to the electrochemically formed Li and the gray domain to the liquid electrolyte. This assignment, however, seems to be, some sort of, lacking of strong evidence for the following reason. The reason is that the authors didn't present the CT results of the Li metal adhered to the stainless steel pin. With this Li metal "reference" missing, it is hard to compare the electrochemically formed Li with the used Li metal "reference" so that one may not know the general X-ray absorption or contrast of the electrochemically formed Li. Can the authors present the CT results containing the Li metal? By the way, what is the distance between the Li metal and the Cu pin? The reviewer noted that the width of the FOV is 0.8 mm, which is smaller that the diameter of the Cu pin (~1.58mm). I am wondering, if the authors can provide the view that contains the side boundary of the Cu pin so that we can have a better idea of the distribution of the liquid electrolyte so that we can know the difference between the region where the Li microstructures are generated (in the presented images) and where the region no Li microstructures are generated? The reason I am asking is that, it seems to the reviewer that the black/dark regions in Fig.2 are, sort of voids that are formed after Li is electrochemically dissolved if we didn't take the electrochemical cycling into consideration.*

**Authors' response:** Distinguishing deposited Li is indeed challenging, but possible as shown by our work. In the experiment we deposit Li on the Cu-pin, thus we are looking at growth of structures at the Cu/liquid electrolyte interface (not Li/liquid electrolyte interface). In fact, there is considerable contrast between these components. One way of assessing this is through the linear attenuation coefficients of X-rays for Li and electrolyte which is shown as a function of wavelength in the figure below, calculated using the database of National Institute of Standards and Technology. At the X-ray energy of the experiment (18 keV) there is more than an order of magnitude difference in the linear attenuation coefficient, with Li-metal having lower attenuation compared to the electrolyte (0.102 $cm^{-1}$ and 1.464 $cm^{-1}$, respectively). This is a comparatively good contrast for X-ray imaging. Note that the attenuation of Cu is very much higher and not at all on this scale. Thus, in an absorption image Cu will be black, electrolyte grey and Li almost white. In the figure below we also show a projection of the cell acquired at low magnification (4x objective, this was done only for purpose of alignment and no tomograms where taken, thus the lower quality of this image) where both the steel pin with Li and the lower Cu pin are visible. In this image one can clearly distinguish the light-colored Li from the electrolyte (grey) and the Cu and steel pins (black). Note that in Figure 3 (previous Figure 2) Li appears as black since the color scales are inverted in the segmentation process. We have added information on the linear attenuation coefficient and the projection of the cell at lower resolution to supporting information, Supplementary Figure 1. In addition, we have added a step by step description on the segmentation of Li in the tomograms in supporting information as Supplementary Figures 13-21.

The distance between the stainless-steel pin and the Cu pin was 2 mm. Thus, when using the high resolution (a 0.33 µm voxel size, 20x objective) to acquire the operando X-ray tomograms, the Li metal adhered to the stainless-steel pin is out of the field of view. In the operando experiments the field of view is 0.8 mm and the cell was not moved in order be able to track the growth of Li structures. Thus, we do not have data showing the boundaries of the Cu pin at these conditions. However, the electrolyte was distributed throughout the cell.

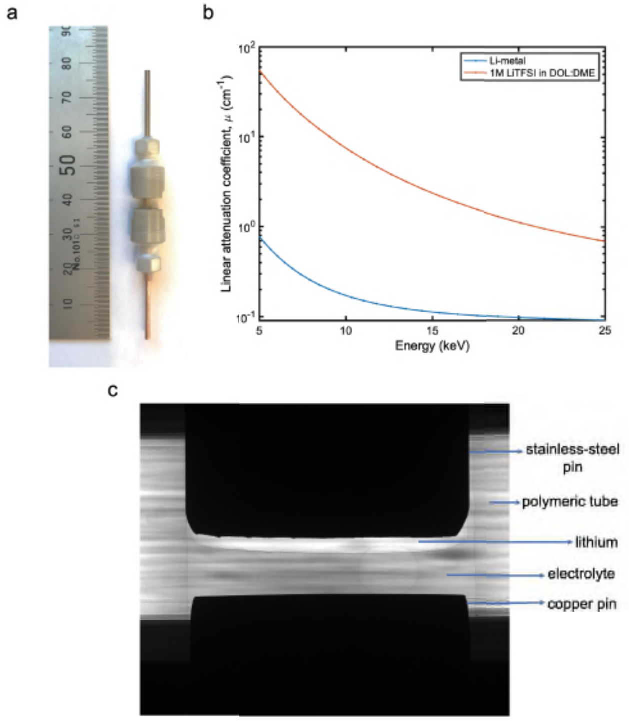


Supplementary Figure 1. Experimental setting for operando X-ray tomographic microscopy. a, Optical image of operando electrochemical cell. b, Calculated X-ray attenuation coefficients of Li metal and electrolyte used in our experiment. The linear attenuation coefficient was calculated based on the electrolyte composition and density with the help of NIST X-Ray Form Factor, Attenuation, and Scattering Tables (https://dx.doi.org/10.18434/T4HS32). c, Representative projection of the operando cell acquired at low resolution.

*2, After the growth and dissolution of Li dendrites part, the authors contained to present the quantitative analysis of the 2D results of the spatial distribution of deposited Li, followed by the qualitative 3D analysis of the evolution of Li microstructure part. Compared with the tedious 2D analysis, I am wondering, why the authors didn't present the vivid and easy-to-comprehend 3D rendering results of the Li microstructure before the 2D analysis result? It seems to the reviewer that the 3D rendering results are much easier for the readers to understand and it will also give an overall view of the evolution of the Li microstructure in a more straightforward way.*

**<u>Authors' response:</u>** Following the suggestion of the reviewer we have reorganized the paper and in the revised manuscript, 3D rendering results and electrochemical data are presented as Figure 2. This is followed by the analysis of vertical slices extracted from the reconstructed 3D tomographic datasets (Figure 3) and the discussion on growth of Li dendrites. Subsequently, the height maps and horizontal slices are discussed. The corresponding texts are moved (but not changed) in the revised manuscript, please see page 6 to page 15.

*3, The regions presented in Figure 2d and Figure 2e are not the same, right? I think it's better to make this clear to the reader.*

**<u>Authors' response:</u>** The slices from the first and second cycle were indeed different, and we agree that this can cause confusion. In the revised manuscript, we have adjusted this and now all slices in Figure 3 on page 9 (previously in Figure 2) are taken from the same position in the 3D tomogram. The position is also marked in a 3D rendering of the data added as Supplementary Figure 5, please see also below. We have also clarified that the slices are from the same position in the 3D datasets on page 8.

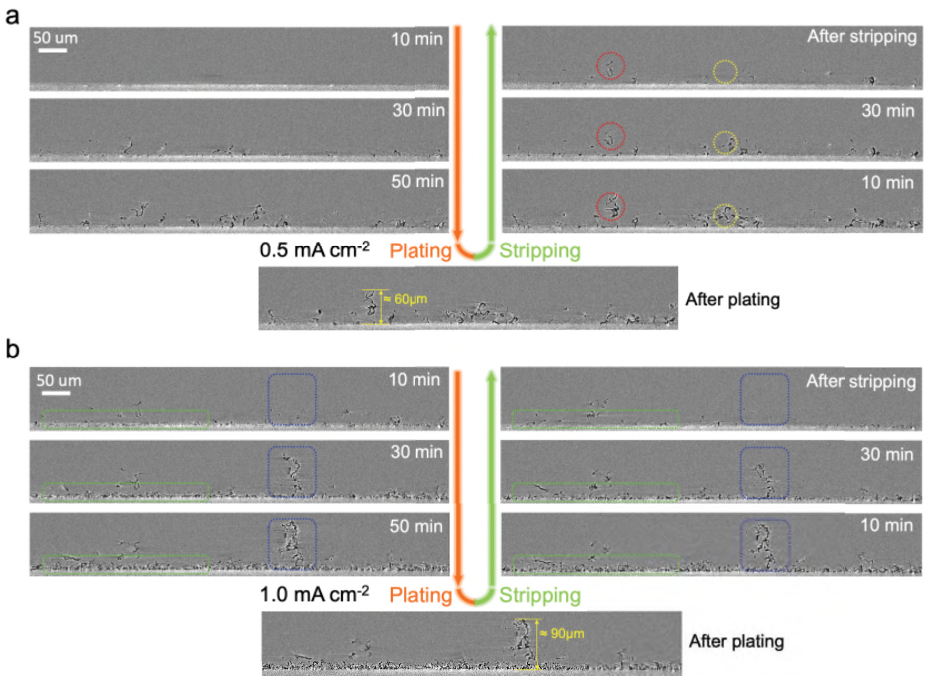


Figure 3 | Operando XTM during plating and stripping at different current densities.

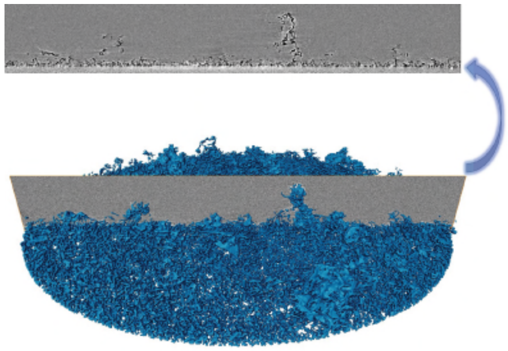

Supplementary Figure 5. Schematic diagram for the extraction of vertical slices from reconstructed 3D tomographic datasets.

*4, The descriptions of the white circles in Figure 3 are not specific and hard to comprehend. On the one hand, the authors highlight two regions in Figure 3a by using two white circles, which are enlarged and shown in Figure 3c and 3d, respectively (it is suggested to mark c,d in the circles in Figure 3a). on the other hand, there are two circles again presented in Figure 3c. But these two circles are marked for different stories, right? Overall, the notations are not logically presented and the corresponding text descriptions are unpleasantly written. This makes the readers hard to understand and follow.*

**<u>Authors' response:</u>** To improve the readability and the presentation of the data in previous Figure 3 (now Figure 5) we have added Roman numbers for each highlighted region, which are also then referred to in the text. The corresponding numbers of notations are added in the revised manuscript, please see pages 11 to 13.

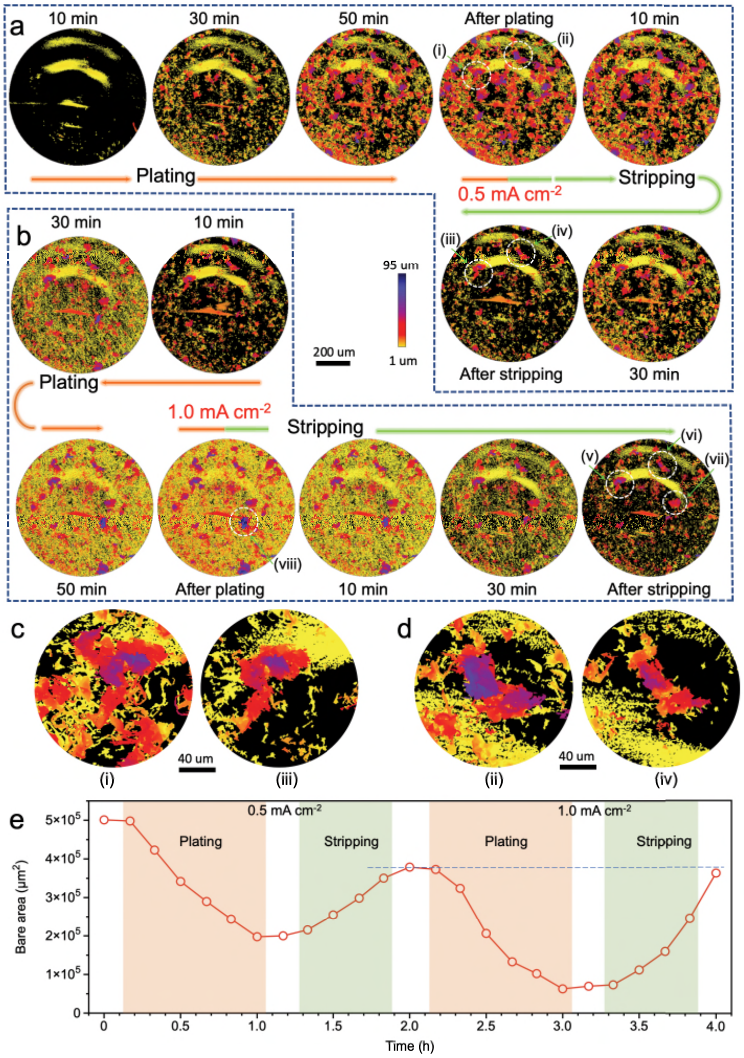


Figure 5 | Height maps of deposited Li at different stages of plating and stripping.

*5, Can't understand the statement on page 8 that "the formation of inactive Li during plating". It is generally widely accepted that the inactive Li become distinguishable during Li stripping process because the inactive Li are not strippable*

*and they remain during stripping process. As a result, we can confirmatively state the remained Li after stripping are inactive Li. However, during plating process with a lot of Li microstructures generated, it is hard to tell which part, if there are, of the Li microstructures are inactive.*

**Authors' response:** We understand the concern raised by the reviewer. Looking in detail into the heightmaps of Li deposited at 0.5 mA $cm^{-2}$ for 40, 50, 60 minutes and after plating one can see that part of the high Li microstructures (blue pixels) already exists on the height map after 50 minutes plating and barely shows any change after that during plating, neither during stripping. This suggests that the disconnection of deposited Li from the electronically conductive network may occur at the early stage of stripping, or even during the plating. We have clarified this in the discussion on the data in the revised manuscript on page 11. We have also added the time series of the close-up region, marked with white circle (i) in Figure 5, as Supplementary Figure 8c.

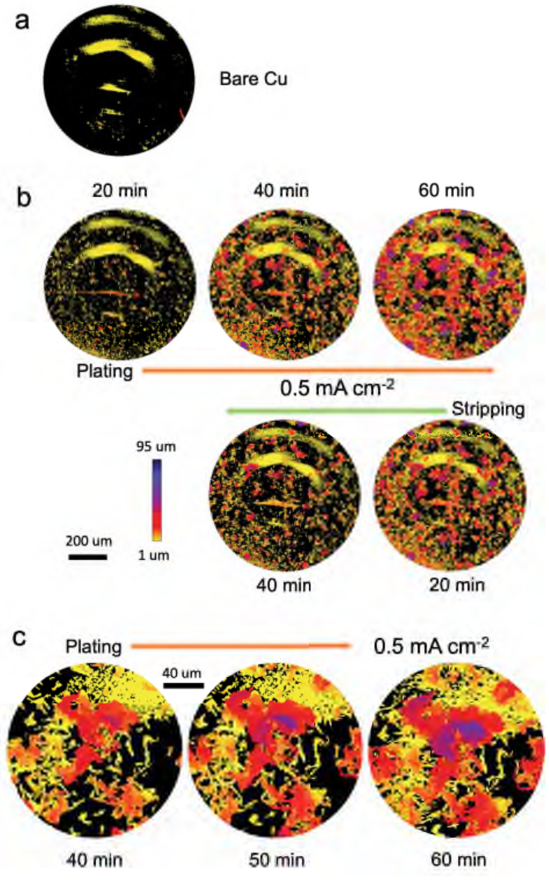


Supplementary Figure 8. Height maps of deposited Li at 0.5 mA $cm^{-2}$. a, Height map of bare Cu substrate. b, Height maps extracted from tomograms taken during plating for 20, 40 and 60 min; during the stripping for 20 and 40 min. c, Close up of Li microstructures marked as cycle (i) in Figure 5a during plating at 0.5 mA $cm^{-2}$.

*6, The colour legend in Figure 3 needs to be changed. If I understand correctly, the dark area in the tomograms are bare Cu, right, which is the lowest height. However, from the colour legend, the dark colour represents the highest, which is 100 um high.*

**Authors' response:** We understand the problem with the color scale in the figure. The black area in the tomograms is bare Cu whereas the highest Li microstructures in fact are dark blue. The color legend is defined by AVIZO and the color legend in use shows the best presentation. To avoid misunderstanding of the color legend we cut it to 95 μm and there is no very dark blue region left there, see Figure 5 (previous Figure 3).

*7, The reviewer can't get the point in Figure 3e where "the amounts of bare area at the same deposition capacity (0.5 mA cm-1 for 1h, 1.0 mA cm-1 for 0.5h, connected by dashed line in Fig. 3e) are very similar, although the architecture of deposited Li (after 1h plating in Fig. 3a and 30 min plating in Fig. 3b) under the two current densities are quite different." What is the message that the authors want to convey?*

**Authors' response:** The point was to emphasize that the amount of bare area is related to the capacity of deposited Li rather than the architecture. However, we see that this discussion is not adding anything, and we have therefore removed this sentence in the revised manuscript and revised the figure by removing that dashed line, see page 14.

*8, the description in figure 4b is not perfect. In the main text, the authors state "In contrast, at high current density in the second cycle there is mainly an increase of the covered area in the slice closest to the substrate (L), whereas the increase further away is smaller than in the first cycle." It is true that in the second cycle, the increase of the covered area closest to the substrate is smaller than that in the first cycle (2L and 3L). However, from Figure 4b, one can clearly note that the increase of the covered area at the distance further away (3L, 4L and 5L) are similar to that in the first cycle. In addition, it seems odds to the reviewer that the authors use "similar or even higher" to describe the amount of extended structures in Figure 4b. How can we correctly understand when two things are "similar" while simultaneous one of these is "even higher"?*

**Authors' response:** We revised the discussion of Figure 6b (previous Figure 4b) following the comments from the reviewer. The revised description is "In contrast, in the second cycle at high current density there is mainly an increase of the covered area in the slice closest to the substrate (L), whereas the increase at 2L-4L is smaller than in the first cycle. At slightly larger distances (5L and 6L) the covered area is similar to that in the first cycle. However, one can also observe that the amount of extended structures is higher far away from the substrate**,** at distances ⩾ 8L (Supplementary Figure 12)." Please see page 15 in the revised manuscript.

*9, in Figure 5c, how the authors calculate the surface area of Li microstructures?*

**Authors' response:** The calculation of the object specific surface of the Li microstructure in Figure 2e (previously in Figure 5c) is carried out using the Object Specific Surface module of AVIZO software in which the object specific surface ($\mu m^2/\mu m^3$) of the 3D case is calculated using the following equation:

$$Object\ Specific\ Surface = \frac{(area3d)}{(volume3d)}$$

$area3d$ is a module in AVIZO that calculates 'the area of the object boundary' which takes into account 'the exposed surfaces of outer voxels'. Thus $area3d$ provides a value in $\mu m^2$ of the exposed Li surface area of binarized Li voxels. $volume3d$ is calculated as the volume of all binarized voxels of Li. We have added this explanation to the methods section on page 19.

***Reviewer #3***

*- What are the noteworthy results?*

*Authors visualized microstructure evolution of lithium plating as a function of time and current density, distinguishing different types of structures and distinguishing the formation of inactive lithium.*

*- Will the work be of significance to the field and related fields? How does it compare to the established literature? If the work is not original, please provide relevant references.*

*This work is significant and will be of interest to many research groups. I agree that it is challenging to do operando experiments with sufficient resolution. However authors need to provide better explanation of novelty of their approach. In the introduction authors stated that it is challenging to visualize and provided references of past work that used heavier elements, formation of cracks or with respect to a separator, claiming there was no direct and continuous tracking of the evolution of Li. What is different in the approach presented here that enabled this team to do what others could not? This is not clear, especially for readers not familiar with the XTM. Authors should better highlight similarities and differences in the approaches and findings between current work and previous reports, and should also should include other citations and their discussion, for example work conducted at the Advanced Photon Source by Seitzman featuring solid electrolyte. Seitzman, N.; Bird, O. F.; Andrykowski, R.; Robbins, S.; Al-Jassim, M. M.; Pylypenko, S., Operando X-ray Tomography Imaging of Solid-State Electrolyte Response to Li Evolution under Realistic Operating Conditions. ACS Applied Energy Materials 2021, 4 (2), 1346-1355.*

**Authors' response:** To highlight our novelty of our work and the approach we revised the last paragraph of introduction on page 4 to highlight the main reasons why we succeed (the high resolution achieved, which is resulted from the configuration of cell, minimization of sample diameter, optimization of contrast between Li and electrolyte and

segmentation of various phases, fast data acquisition). The mentioned previous works were also added in the introduction, Ref. 42 in the revised manuscript.

*- Does the work support the conclusions and claims, or is additional evidence needed?*

*This paper is written well, and all conclusions are supported by the data. Figure 2 needs additional markers and its discussion should be revised to make it clear what area in Fig 2 is being discussed. Fig. 2 shows several regions of interest-one area was highlighted in yellow circle, another in red oval, and third was marked with green rectangle. In some places authors referred to specific area and how it was marked, but in some places, they referred to area without indicated how it was marked (for example green area). Also, there was additional area of interest discussed, with mossy and needle-like structure, but the area is not marked in the Fig 2.*

**Authors' response:** To increase the readability of the discussion of figure 2 (figure 3 in the revised manuscript) the highlighted regions on vertical slices are explicitly referred to in the text on page 8 to page 10 and all specific regions discussed are now marked.

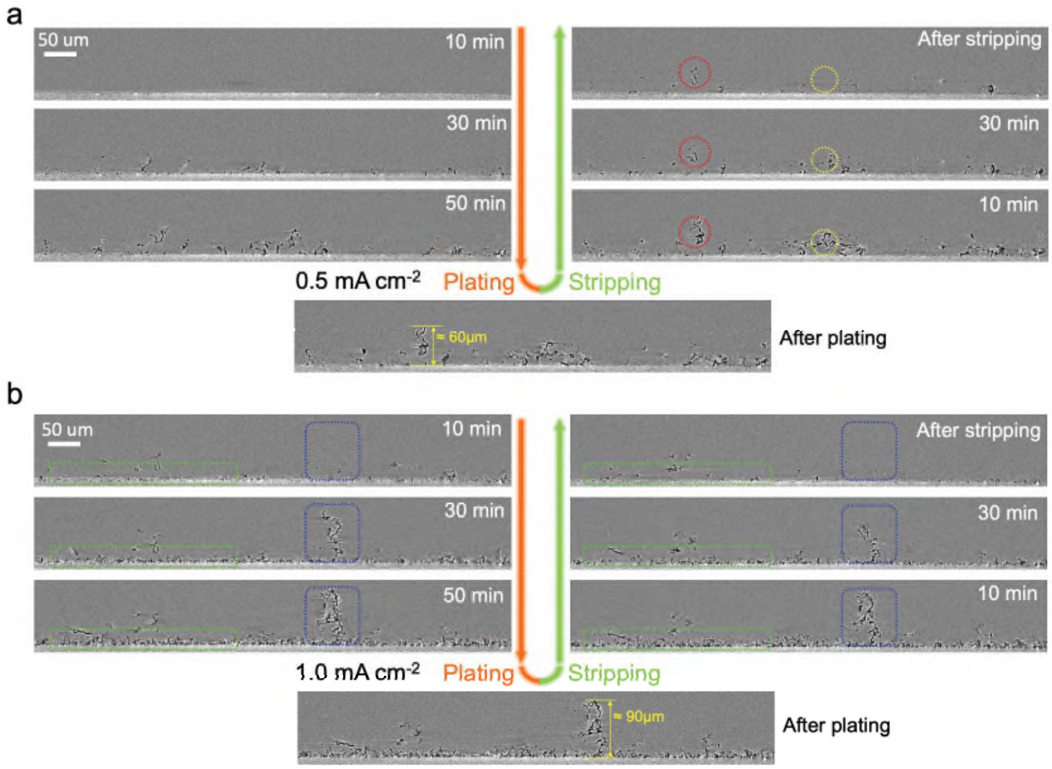


Figure 3 | Operando XTM during plating and stripping at different current densities.

*- Are there any flaws in the data analysis, interpretation and conclusions? - Do these prohibit publication or require revision?*

*Authors should make a stronger statement of the impact of this study on design of ideal structures.*

**Authors' response:** To make a stronger statement of the impact of this study we have added discussion on page 16.

*- Is the methodology sound? Does the work meet the expected standards in your field?*

*Yes*

*- Is there enough detail provided in the methods for the work to be reproduced?*

*Yes*

**REVIEWERS' COMMENTS**

Reviewer #1 (Remarks to the Author):

The authors have carefully, thoughtfully and adequately addressed all of the concerns raised in my original review.

Reviewer #2 (Remarks to the Author):

since the authors have addressed the reviewer's comment, it seems to the reviewer that the manuscript is ready for acceptance.
